# A Flavor-Oriented Multi-Criteria Screening Strategy Identifies Indigenous Lactic Acid Bacteria and Yeasts for Improving Fermented Camel Milk Quality

**DOI:** 10.3390/foods15203610

**Published:** 2026-10-10

**Authors:** Yabin Dai, Juan Ma, Shengchang Zhou, Haitao Yue, Tong Shen, Dilireba Shataer, Luoyizha Wahafu

**Affiliations:** 1 College of Smart Agriculture (Research Institute), Xinjiang University, Urumqi 830017, China; dreamchaseryb@163.com (Y.D.); mj012789@163.com (J.M.); zhoushengchangsd@163.com (S.Z.); dlrb_shataer@2021.xju.edu.cn (D.S.); 2 College of Life Science and Technology, Xinjiang University, Urumqi 830017, China; yuehaitao@tsinghua.org.cn (H.Y.); st@xju.edu.cn (T.S.)

**Keywords:** fermented camel milk, indigenous strains, volatile flavor compounds, HS-SPME-GC-MS, relative odor activity value

## Abstract

The inconsistent flavor of traditionally fermented camel milk (CM) limits its standardization and broader consumer acceptance. A total of 198 lactic acid bacteria (LAB) strains and 120 yeast strains were isolated and identified from traditionally fermented camel milk samples collected in Xinjiang, China. Following taxonomic classification and preliminary screening of the isolates, 39 LAB strains (designated DL1–DL39) and 25 yeast strains (designated DY1–DY25) were selected for further investigation. LAB were first screened for acidification capacity and proteolytic activity, and yeasts were screened for aroma and gas production and acid tolerance; promising strains were then refined by integrating textural properties, E-nose, E-tongue, and sensory evaluation, and their key volatiles were characterized by gas chromatography–mass spectrometry (HS-SPME-GC-MS) combined with relative odor activity values (rOAVs). Two optimal LAB strains, DL19 (*Lacticaseibacillus paracasei*) and DL29 (*Lactiplantibacillus plantarum*), and one superior yeast strain, DY3 (*Kluyveromyces marxianus*), were finally selected. CM fermented by strain DL19 achieved the highest overall sensory acceptability, whereas that fermented by strain DL29 received a significantly higher odor score. HS-SPME-GC-MS analysis identified the compounds responsible for the creamy, buttery, and floral notes in the fermented products, namely 2,4-undecadienal, (2E,4Z)-2,4-decadienal, and *δ*-nonalactone. DY3 produced the richest odor-active substances, with 2-phenylethanol and γ-undecalactone endowing CM with distinct floral, fruity, and creamy flavors. WGS analysis provided a preliminary assessment of safety-related genomic features of DL19, DL29, and DY3. In conclusion, these indigenous strains show great potential as starter or adjunct cultures for precise flavor regulation of fermented CM. The established multi-index evaluation system effectively supports the standardized production of high-quality fermented CM, facilitating the high-value sustainable utilization of underutilized regional CM resources within the future food supply chain.

## 1. Introduction

Dairy products have long attracted considerable research interest owing to their nutritional value and diverse sensory profiles. Products such as yogurt, cheese, butter, and kefir have become integral components of human diets worldwide. Among the major milk-producing livestock species—cattle, buffalo, camels, goats, and sheep—camels are uniquely adapted to arid and semi-arid environments, where they can maintain relatively stable milk production under harsh climatic conditions, establishing them as a vital dairy resource across parts of the Middle East, Africa, and Asia [1,2]. Camel milk (CM) is nutritionally distinctive, being characterized by lower cholesterol content and high concentrations of essential minerals and vitamins, including vitamins A, C, D, and E. In addition, CM contains elevated levels of bioactive protective proteins, including lysozyme, immunoglobulins, lactoferrin, and lactoperoxidase, which collectively contribute to its unique functional properties and potential physiological benefits [3,4]. Despite these recognized nutritional merits, the market acceptance of CM remains limited, largely owing to its characteristic salty, goaty, and fishy off-notes that reduce consumer acceptance and restrict its broader utilization in the dairy industry. Improving the flavor profile of CM and enhancing its overall palatability therefore represent key priorities for advancing CM-based processing and product development.

Microbial fermentation is widely applied to improve the flavor, consistency, and nutritional quality of dairy products. During fermentation, lactic acid bacteria (LAB) and yeasts generate various flavor-active compounds—including organic acids, aldehydes, alcohols, esters, and ketones—primarily through lactose metabolism, proteolysis, lipolysis, and amino acid catabolism [5]. Traditional fermented CM products (e.g., shubat, chal, and suusac) typically rely on spontaneous fermentation by indigenous microorganisms from the raw milk and processing environment, which impart distinct regional sour and alcoholic notes. These regional flavor characteristics are largely determined by the composition of the indigenous microbial community. Bilal et al. [6] reported significant differences in volatile organic compounds among shubat samples from different regions; the Turkestan region had the highest proportions of alcohol and acids, while the Almaty and Kyzylorda regions had higher concentrations of esters. They attributed these differences to variations in spontaneous fermentation processes resulting from differences in microbial communities across regions. Therefore, utilizing defined starter cultures is essential to achieve controlled fermentation and ensure consistent product quality.

Currently, flavor and texture improvement in yogurt primarily relies on LAB starter cultures, particularly *Streptococcus thermophilus* and *Lactobacillus delbrueckii* subsp. *bulgaricus*, which are central to enhancing acidification efficiency, stabilizing the fermentation process, and strengthening gel structure [7]. However, the direct application of conventional bovine milk (BM) starter cultures to the CM matrix may not be fully appropriate and may prove insufficient in addressing the inherent flavor limitations of CM. Galeboe et al. [8] produced CM yogurt using the commercial thermophilic yogurt culture YF-L811, composed of *L. delbrueckii* subsp. *bulgaricus* and *S. thermophilus*, together with texture- and flavor-improving ingredients. Nevertheless, sensory evaluation revealed that BM yogurt remained preferred over the CM product, which received lower scores for aroma, mouthfeel, and overall acceptability. Microstructural and rheological studies have further confirmed that BM-adapted strains, including *S. thermophilus*, *L. delbrueckii* subsp. *bulgaricus*, *Lactobacillus helveticus*, *Lacticaseibacillus casei*, and *Lactiplantibacillus plantarum* consistently underperform in CM, exhibiting reduced bacterial viability, altered acidification kinetics, and more pronounced proteolysis, yielding gel networks that are looser, more porous, and rheologically weaker than those formed in BM [9]. However, Soleymanzadeh et al. [10] isolated indigenous LAB from chal and used them to ferment both CM and BM; CM fermented with *Leuconostoc lactis* SM10 outperformed the fermented BM in consumer acceptance. Therefore, screening for flavor-enhancing starter cultures suited to the CM matrix is the focus of our current work.

Several studies have identified indigenous LAB in traditional CM across various geographical regions, including Iran, Kazakhstan, East Africa, and Northwest China (predominantly Xinjiang and Inner Mongolia), with the dominant microbiota comprising *Lactobacillus*, *Lactococcus*, *Streptococcus*, *Weissella*, and *Enterococcus* [11,12,13,14,15]. However, most previous studies have focused on isolating and identifying LAB strains and testing their probiotic potential in vitro. Much less attention has been paid to indigenous yeast strains and their role in improving CM flavor. This gap points to the need for a systematic, flavor-directed screening strategy for indigenous strains. A practical approach should evaluate both the acidification capacity of candidate strains (to confirm matrix compatibility and fermentation activity) and their ability to improve texture and sensory characteristics. In this study, we isolated LAB and yeasts from traditionally fermented CM using systematic screening based on fermentation performance and flavor-forming ability. We then compared how different strains affected CM flavor and quality, using proteolytic activity, acid tolerance, texture analysis, electronic nose (E-nose), electronic tongue (E-tongue), sensory evaluation, headspace solid-phase microextraction coupled with gas chromatography–mass spectrometry (HS-SPME-GC-MS), and whole-genome sequencing (WGS) as key evaluation tools.

## 2. Materials and Methods

### 2.1. Materials

Fifteen traditionally fermented CM samples for strain isolation were collected from five regions of Xinjiang, China, including Ili, Urumqi, Altay, Changji, and Aksu. Three samples were collected from each region, and each sample was obtained from a different local household producer (Table S1). According to information provided by the producers, the CM was traditionally fermented without commercial starter cultures, relying on indigenous microorganisms. Fresh CM used for the fermentation experiments was obtained from Gejiagou Camel Farm (Urumqi, Xinjiang, China) and was from the same batch; the relevant components are shown in Table 1. All samples were transported on dry ice and stored long-term at −80 °C for subsequent analysis. Lactic acid was obtained from Xinbot Chemical Co., Ltd. (Tianjin, China), and borax was obtained from Zhiyuan Chemical Reagents Co., Ltd. (Tianjin, China). Sodium dodecyl sulfate (SDS), o-phthalaldehyde (OPA), and 1,4-dithiothreitol (DTT) were purchased from Solarbio Science & Technology Co., Ltd. (Beijing, China).

### 2.2. Isolation and Preliminary Identification of Strains

**Figure 1:**
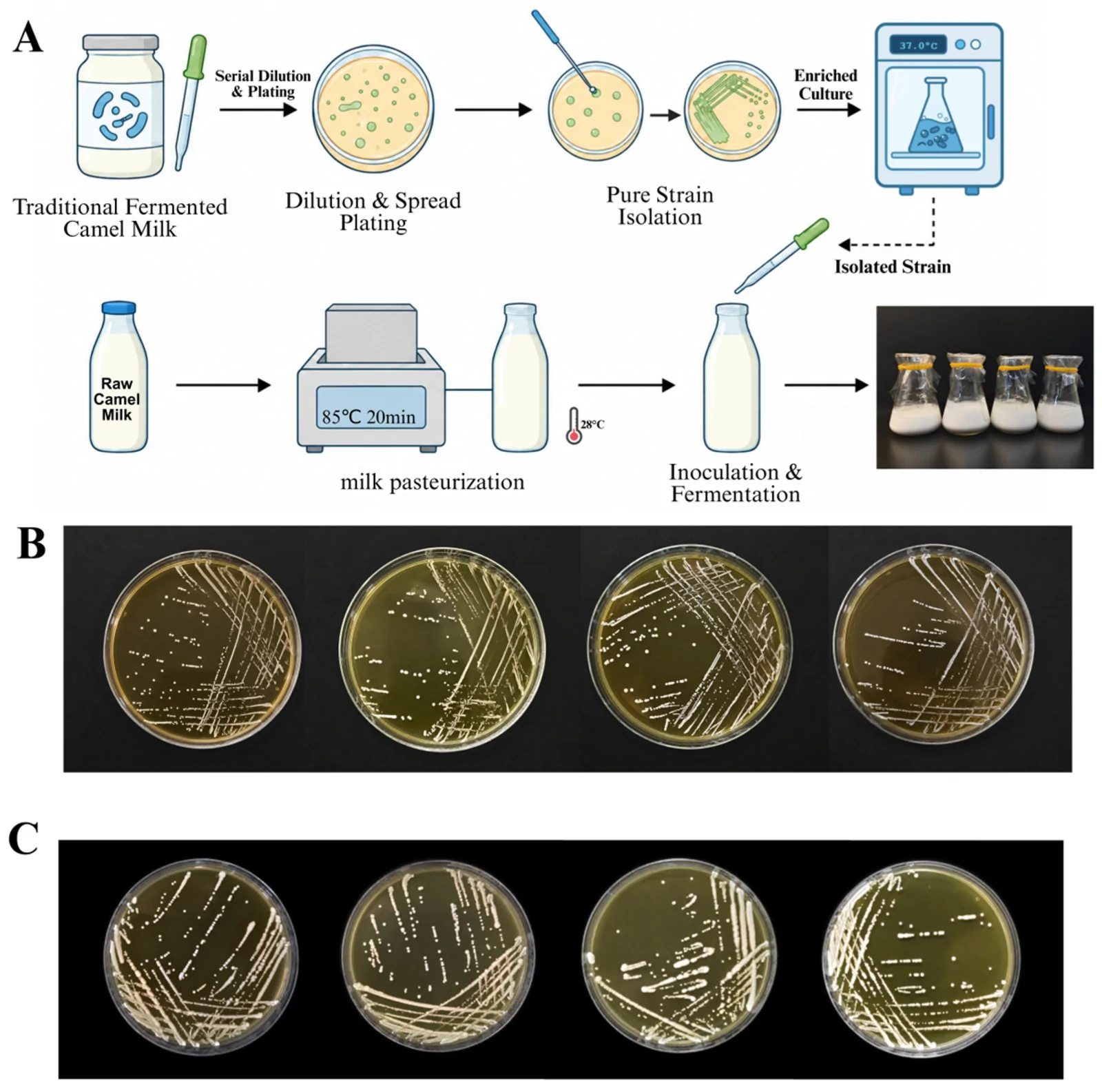
Isolation, screening, and fermentation process of LAB and yeast strains in traditionally fermented CM. (**A**) Schematic diagram of the isolation, purification, and fermentation processes. (**B**) Colony morphologies of selected purified LAB isolates. (**C**) Colony morphologies of selected purified yeast isolates.

For each traditionally fermented CM sample, 10 mL was inoculated separately into 40 mL of MRS broth (for LAB) or YPD broth (for yeasts) and incubated aerobically at 37 °C or 28 °C for 12–18 h, respectively. The cultures were serially diluted in 0.85% (*w*/*v*) sterile saline, and 200 μL of appropriate dilutions were spread onto the corresponding agar plates. MRS plates for LAB isolation were incubated under aerobic conditions at 37 °C, whereas YPD plates for yeast isolation were incubated under aerobic conditions at 28 °C for 48 h. Aerobic cultivation was used to facilitate the selection of strains potentially suitable for industrial fermentation, where strictly anaerobic conditions are generally not maintained. Moreover, aerobic cultivation may favor the growth and recovery of relatively oxygen-tolerant LAB and may therefore introduce some selection bias into the recovered isolates. After incubation under the corresponding conditions for 48 h, morphologically distinct colonies were subcultured to obtain pure isolates (Figure 1). All isolates were stored at −80 °C in broth containing 25% (*v*/*v*) glycerol.

### 2.3. Molecular Identification by 16S rRNA and 26S rDNA Gene Sequencing

Genomic DNA was extracted from all isolates using bacterial (Tingen Biotech, Beijing, China) and fungal (Sangon Biotech, Shanghai, China) genomic DNA extraction kits. The 16S rRNA gene of LAB and the D1/D2 region of the 26S rDNA of yeasts were amplified using the universal primer pairs 27F/1492R and NL1/NL4, respectively [16]. Amplicons were sequenced bidirectionally by Genewiz (Suzhou, China). Taxonomic identification was performed by comparing the obtained sequences against the NCBI GenBank database using BLAST 2.2.24. The sequence length, percentage identity, query coverage, closest reference sequence, and GenBank accession number for each representative isolate are provided in Supplementary Table S2.

For phylogenetic analysis, the obtained sequences and selected reference sequences retrieved from GenBank were aligned using MUSCLE implemented in MEGA 12. Phylogenetic trees were reconstructed using the neighbor-joining method based on p-distance, with 1000 bootstrap replicates used to evaluate branch support. Gaps and missing data were treated using the partial-deletion option with a site coverage cutoff of 95%. The final alignments contained 1341 nucleotide positions for the LAB dataset and 531 nucleotide positions for the yeast dataset. Because the neighbor-joining analysis was based on p-distance, no additional nucleotide substitution model was applied.

### 2.4. Preparation of Fermented CM

Fresh CM was pasteurized at 85 °C for 20 min and cooled. LAB and yeast strains were harvested by centrifugation at 4000× *g*, washed twice, and resuspended in sterile saline. The standardized LAB and yeast inocula were added to pasteurized CM at 3% (*v*/*v*) and 2% (*v*/*v*), respectively, with inoculum concentrations of 10^8^ CFU/mL for LAB and 10^6^ CFU/mL for yeasts (Figure 1A). Each strain was evaluated in three independent fermentation replicates. Fermentation was conducted at 37 °C or 28 °C for 24 h, followed by maturation at 4 °C for 12 h.

### 2.5. Preliminary Screening of LAB

#### 2.5.1. pH and Titratable Acidity

The pH of each fermented CM sample was measured directly using a calibrated pH meter. Titratable acidity (TA °T) was determined by NaOH titration following a standard procedure [17]. Briefly, 10 g of each sample was mixed with phenolphthalein as an indicator and titrated with 0.1 mol·L^−1^ NaOH solution until a faint pink color persisted for 30 s. TA was calculated according to the following formula:

$$\mathrm{TA}\;({}^{\circ}T)=\frac{V_{NaOH}\times C_{NaOH}\times 100}{M_{Sample}\times 0.1}$$

where V_NaOH_ is the volume of NaOH consumed (mL), C_NaOH_ is the molar concentration of NaOH (mol·L^−1^), and M_Sample_ is the mass of sample used for titration (g).

#### 2.5.2. Proteolytic Activity

Proteolytic activity was determined using the OPA method [18]. A total of 5 mL of fermented milk was mixed with an equal volume of 15% (*w*/*v*) TCA to precipitate proteins, followed by centrifugation (9000× *g*, 15 min, 4 °C) and filtration (0.45 μm). Then, 100 μL of filtrate was reacted with 3 mL of OPA reagent for 2 min in the dark. The absorbance was measured at 340 nm. Proteolytic activity was calculated as the increase in OPA-reactive free amino groups relative to the unfermented control.

### 2.6. Preliminary Screening of Yeast

#### 2.6.1. Gas Production and Aroma Production

(1) Gas production was evaluated as a preliminary semi-quantitative screening parameter. For each yeast strain, three independent fermentation replicates were prepared in 15 mL sealed containers containing 8 mL of CM and incubated at 28 °C for 24 h. Uninoculated CM was included as the control. Gas production was assessed immediately after opening the containers according to visible effervescence, foam formation, and pressure release. The response was classified into four levels: none, no visible bubbles or gas release; weak, only a small number of bubbles; moderate, obvious and sustained bubbling; and strong, rapid bubbling accompanied by marked pressure release. The same criteria were applied to all samples and replicates.

(2) Aroma production was evaluated immediately after fermentation by 12 evaluators from a food science laboratory who were familiar with the sensory characteristics of fermented dairy products. Each yeast strain was evaluated using three independent fermentation replicates, and all replicates were assessed by all evaluators. The samples were presented without information on strain identity, so that the evaluators were blinded to the strain being assessed. Each evaluator independently assigned the predominant odor characteristic of each sample to one of four categories: (i) lactic acid flavor, characterized predominantly by sour and fermented-milk notes; (ii) ethanol flavor, characterized predominantly by alcoholic notes; (iii) no obvious characteristic flavor; or (iv) off-flavor, including rancid and putrid. When more than one odor characteristic was perceived, classification was based on the predominant odor. The final category assigned to each sample was determined by majority voting among the 12 evaluators [19].

#### 2.6.2. Acid Tolerance

Acid tolerance was evaluated by inoculating 2% (*v*/*v*) yeast suspension (10^6^ CFU/mL) into YPD broth adjusted to pH 2.5, 3.0, 3.5, 4.0, 4.5, and 5.0 with 1 mol·L^−1^ lactic acid, followed by incubation at 28 °C for 24 h. Growth was monitored by measuring optical density at 600 nm (OD_600_) [16].

### 2.7. Syneresis

Syneresis was determined according to the method described by Bratkič et al. [20], with slight modifications. A fermented CM sample of known mass (M_0_, g) was transferred to a pre-weighed centrifuge tube and centrifuged at 4000× *g* for 15 min at 4 °C. The supernatant (expelled whey) was carefully removed, and the mass of the retained gel was recorded as M_1_ (g). Syneresis was calculated according to the following equation:

$$Syneresis\left(\%\right)=\frac{{M_{0}-M}_{1}}{M_{0}}\times 100\%$$

where M_0_ is the initial mass of the fermented milk sample (g), and M_1_ is the mass of the gel sediment retained after centrifugation (g).

### 2.8. Particle Size Measurement

Particle size distribution was determined by dynamic light scattering (DLS) using a Zetasizer Nano ZS90 analyzer (Malvern Instruments, Malvern, UK). Before analysis, each sample was diluted 200-fold with deionized water and equilibrated for 3 min at 25 °C. Deionized water was used as the dispersant, and its refractive index was set to 1.33 in the instrument software. Measurements were performed at 25 °C, and each sample was measured in triplicate. Particle-size distributions were analyzed on an intensity basis, and the polydispersity index (PDI) was obtained from the DLS measurements.

### 2.9. Apparent Viscosity and Viscoelastic Properties

The rheological properties of fermented CM were measured at 25 °C using a rotational rheometer (RH20, Shanghai Bosin Industrial Development Co., Ltd., Shanghai, China). The samples were placed between parallel plates with a fixed gap of 1.0 mm. Apparent viscosity was measured over a shear rate range of 0.01–100 s^−1^. The viscoelastic properties of the samples were evaluated by conducting frequency sweep tests over a range of 0.1–10 Hz to determine their storage and loss behavior [21,22].

### 2.10. E-Nose and E-Tongue Analysis

Volatile profiles of fermented CM samples were characterized using an electronic nose (C-PEN3, Beijing Enovel Technology Development Co., Ltd., Beijing, China). For each measurement, 10 mL of sample was transferred into a sealed vial and equilibrated at 25 °C for 30 min. Before sample analysis, the sensor chamber was purged with clean air for 100 s and a baseline response was recorded. The headspace gas was then introduced into the sensor chamber for 80 s at a flow rate of 400 mL·min^−1^. After each measurement, the sensor array was cleaned with clean air for 100 s before analysis of the next sample. Sensor responses were recorded continuously, and the mean response over the final 5 s of the stable plateau was used for subsequent analysis. Responses were expressed as the conductivity ratio G/G_0_. Each sample was analyzed in triplicate [23].

Taste profiles were evaluated using an electronic tongue system (402B-C, Beijing Enovel Technology Development Co., Ltd., Beijing, China) for eight taste attributes: sourness, bitterness, astringency, after-astringency, after-bitterness, umami, richness, and saltiness. Before analysis, fermented CM samples were diluted 1:1 (*v*/*v*) with deionized water and centrifuged at 6000× *g* for 10 min at 4 °C to remove the fat layer. Measurements were conducted at 25 °C. The sensors were cleaned between samples using the standard cleaning procedure provided by the instrument. Three independent fermentation replicates were analyzed for each treatment. Taste responses were expressed as relative differences from the unfermented CM control [24].

### 2.11. Sensory Evaluation

The sensory evaluation of fermented CM was conducted by a panel of 12 graduate students majoring in food science who were familiar with the sensory characteristics of fermented dairy products (aged 22–29). Samples were coded and presented at room temperature (25 °C) in randomized order. Each assessor randomly selected the coded samples for evaluation, and each sample was served at a volume of 20 mL. Each assessor independently evaluated color, consistency, odor, and taste using a 6-point sensory scoring scale ranging from 0 (least desirable) to 5 (most desirable). Purified water was provided for palate cleansing between samples. All samples were evaluated within a single sensory evaluation session, and all assessors evaluated all samples. Each strain was represented by three independent fermentation replicates [25]. The study focused on flavor-oriented strain screening rather than consumer acceptance; predefined study-specific weighting factors (Fi) were applied to the four sensory attributes, with greater emphasis placed on odor and taste than on color and consistency. The weighting factors for color, consistency, odor, and taste were 0.4, 0.4, 1.2, and 1.2, respectively, corresponding to a relative weighting ratio of 1:1:3:3 (Table 2). Accordingly, odor and taste accounted for 75% of the total weighting, whereas color and consistency accounted for 25%. These coefficients were used as study-specific screening weights and were not intended to represent validated consumer-preference coefficients or a standardized sensory acceptance scale. The weighted sensory screening score (S) was calculated as follows:

S = Σ (mean attribute score × Fi)

### 2.12. HS-SPME-GC-MS Analysis

Fermented CM samples were stored at −80 °C and thawed on ice before analysis. After vortex mixing, 0.2 mL of each sample was transferred into a 20 mL headspace vial, followed by the addition of 0.2 g NaCl and 20 μL of 3-hexanone-2,2,4,4-d4 (10 μg/mL) as the internal standard. The vial was immediately sealed with a crimp cap fitted with a TFE silicone septum. Samples were equilibrated with agitation at 60 °C for 5 min. A 120 μm DVB/CWR/PDMS SPME Arrow (Agilent, Folsom, CA, USA) was then exposed to the headspace at 60 °C for 15 min. After extraction, the SPME Arrow was immediately transferred to the GC inlet and thermally desorbed at 250 °C for 5 min.

Volatile compounds were analyzed using an Agilent 8890 gas chromatograph coupled to an Agilent 7000D mass spectrometer and equipped with a DB-5MS capillary column (30 m × 0.25 mm, 0.25 μm film thickness). Helium was used as the carrier gas at a constant flow rate of 1.2 mL·min^−1^. The oven temperature was held at 40 °C for 3.5 min, increased to 100 °C at 10 °C/min, then to 180 °C at 7 °C/min, and finally to 280 °C at 25 °C/min, followed by a 5 min hold. Electron ionization was performed at 70 eV in selected ion monitoring mode. The ion source, quadrupole, and transfer line temperatures were maintained at 230, 150, and 280 °C, respectively.

Volatile compounds were identified by comparison of their retention times, retention indices, and quantifier and qualifier ions with an in-house database established from reference standards and the literature data. Each compound was monitored using one quantifier ion and two to three qualifier ions. Semi-quantitative concentrations were calculated relative to the internal standard according to the following equation [26]:

$$X_{i}=\frac{V_{s}\times C_{s}}{V}\times \frac{I_{i}}{i_{s}}\times 10^{-3}$$

where *X_i_* is the concentration of compound i in the sample (μg/mL); *Vs* is the volume of the internal standard added (μL); *Cs* is the concentration of the internal standard (μg/mL); *V* is the volume of the sample (mL); *Is* is the peak area of the internal standard; and *I_i_* is the peak area of compound i in the sample.

### 2.13. Relative Odor Activity Value Analysis

The relative odor activity value (rOAV) was calculated to estimate the contribution of individual volatile compounds to the aroma profile according to the following equation [27]:

$${rOAV}_{i}=\frac{C_{i}}{T_{i}}$$

where *Ci* is the semi-quantitative concentration of the compound, and *Ti* is its odor threshold. Concentration and threshold values were expressed in consistent units before calculation. Compounds with rOAV ≥ 1 were considered to have a direct contribution to the aroma profile.

### 2.14. Genome Analysis and Preliminary Safety Assessment

Genomic DNA was extracted from harvested cells using a Bacteria DNA Kit (OMEGA) for the bacterial strains and a Fungi DNA Kit (OMEGA) for the yeast strain, according to the manufacturer’s instructions. The purified genomic DNA was subsequently subjected to quality assessment and quantified using a TBS-380 fluorometer (Turner BioSystems Inc., Sunnyvale, CA, USA). DNA samples with an OD_260_/_280_ ratio of 1.8–2.0 and a total DNA amount >6 μg were used for library preparation. For each strain, at least 1 μg of genomic DNA was used to construct a paired-end sequencing library with an insert size of approximately 400 bp. Genomic DNA was fragmented using a Covaris system, followed by end repair, 3′ A-tailing, adapter ligation, fragment selection, PCR enrichment, and library quality assessment. Qualified libraries were subjected to paired-end sequencing (2 × 150 bp) by Shanghai Biozeron Biotechnology Co., Ltd. (Shanghai, China). The extraction and library-construction procedures followed the protocols provided by the sequencing company.

Raw paired-end reads were trimmed and quality-filtered using Trimmomatic [28] v0.36 with the parameters SLIDINGWINDOW:4:15 and MINLEN:75. The resulting clean reads were used for de novo genome assembly. According to the sequencing provider protocols, the bacterial genomes were assembled using ABySS v2.2.0, whereas the yeast genome was assembled using ABySS v2.0.2 [29]. Multiple k-mer values were evaluated to obtain the optimal assemblies, after which GapCloser was applied to fill remaining local gaps in the draft genome assemblies.

For the bacterial strains, protein-coding genes were predicted using GeneMark v4.17 [30]. Predicted bacterial proteins were compared against the Comprehensive Antibiotic Resistance Database (CARD; https://card.mcmaster.ca/, accessed on 3 October 2026) and the Virulence Factor Database (VFDB; https://www.mgc.ac.cn/VFs/, accessed on 3 October 2026) to identify antimicrobial-resistance- and virulence-associated homologs, respectively.

For the yeast strain, repetitive sequences were identified using RepeatMasker v4.0.7 (https://www.repeatmasker.org/), and protein-coding genes were predicted using AUGUSTUS (https://bioinf.uni-greifswald.de/augustus/, accessed on 3 October 2026) [31]. Fungal pathogenicity-related annotations were evaluated using PHI-base (https://phi5.phi-base.org/) and the Database of Fungal Virulence Factors (DFVF; http://sysbio.unl.edu/DFVF/, accessed on 3 October 2026).

### 2.15. Statistical Analysis

All experiments were performed in triplicate, encompassing independent biological fermentation batches and repeated technical instrumental measurements, with results expressed as mean ± standard deviation. Data were analyzed by one-way analysis of variance using SPSS 26.0 (IBM Corp., Armonk, NY, USA), and mean separations were determined using Duncan’s multiple range test with significance defined at *p* < 0.05.

For volatile flavor analysis, semi-quantitative concentrations of volatile compounds calculated relative to the internal standard were used as the input abundance matrix. Pearson correlation coefficients were calculated using the cor function in R version 4.1.2. Principal coordinate analysis (PCoA) was performed in R version 4.1.2 using the Bray–Curtis distance to visualize differences in volatile composition among samples.

Orthogonal partial least squares discriminant analysis (OPLS-DA) was performed using MetaboAnalystR version 1.0.1. Before OPLS-DA, the volatile-abundance data were log_2_-transformed and subjected to unit-variance scaling. The OPLS-DA models used one predictive component, and the first orthogonal component was used together with the predictive component for two-dimensional score plots. Model validation was performed using 7-fold cross-validation. Model performance was evaluated using R^2^X, R^2^Y, and Q^2^, representing the explained variance of the X and Y matrices and the predictive ability of the model, respectively. Model robustness and potential overfitting were assessed using 200 permutation tests with randomly permuted class labels.

Variable importance in projection (VIP) values were obtained from the OPLS-DA models, and VIP ≥ 1 was used as the multivariate screening threshold. For pairwise comparisons, differential volatile compounds were defined using the combined criteria of VIP ≥ 1, *p* < 0.05, and |log_2_FC| > 1. Log_2_ fold changes were calculated for comparisons between the fermented CM samples and the unfermented CM control. Volcano plots were generated based on fold changes and statistical significance. Graphs were prepared using R and Origin 2024 (OriginLab Corp., Northampton, MA, USA).

## 3. Results and Discussion

### 3.1. Isolation and Identification of Fermentation Strains

A total of 198 LAB and 120 yeast strains were isolated from traditionally fermented CM samples collected from five geographically distinct regions. In total, 16S/26S rRNA gene amplification and sequencing analyses were performed. All sequences were queried against the NCBI GenBank database using BLAST for taxonomic assignment. Following species-level identification, isolates assigned to the same taxonomic group were grouped for representative selection. Geographic origin was used as a prioritization criterion rather than as a strict one-isolate-per-region rule. Within each taxonomic group, isolates originating from different geographic regions were preferentially retained to improve geographic representation and reduce overrepresentation of isolates from the same source region. As a result, 39 representative LAB isolates and 25 yeast isolates were retained for subsequent fermentation screening. These isolates were designated DL1–DL39 and DY1–DY25, respectively, and their taxonomic identities are summarized in Supplementary Figures S1 and S2 and Table S2.

### 3.2. Preliminary Screening of LAB

**Figure 2:**
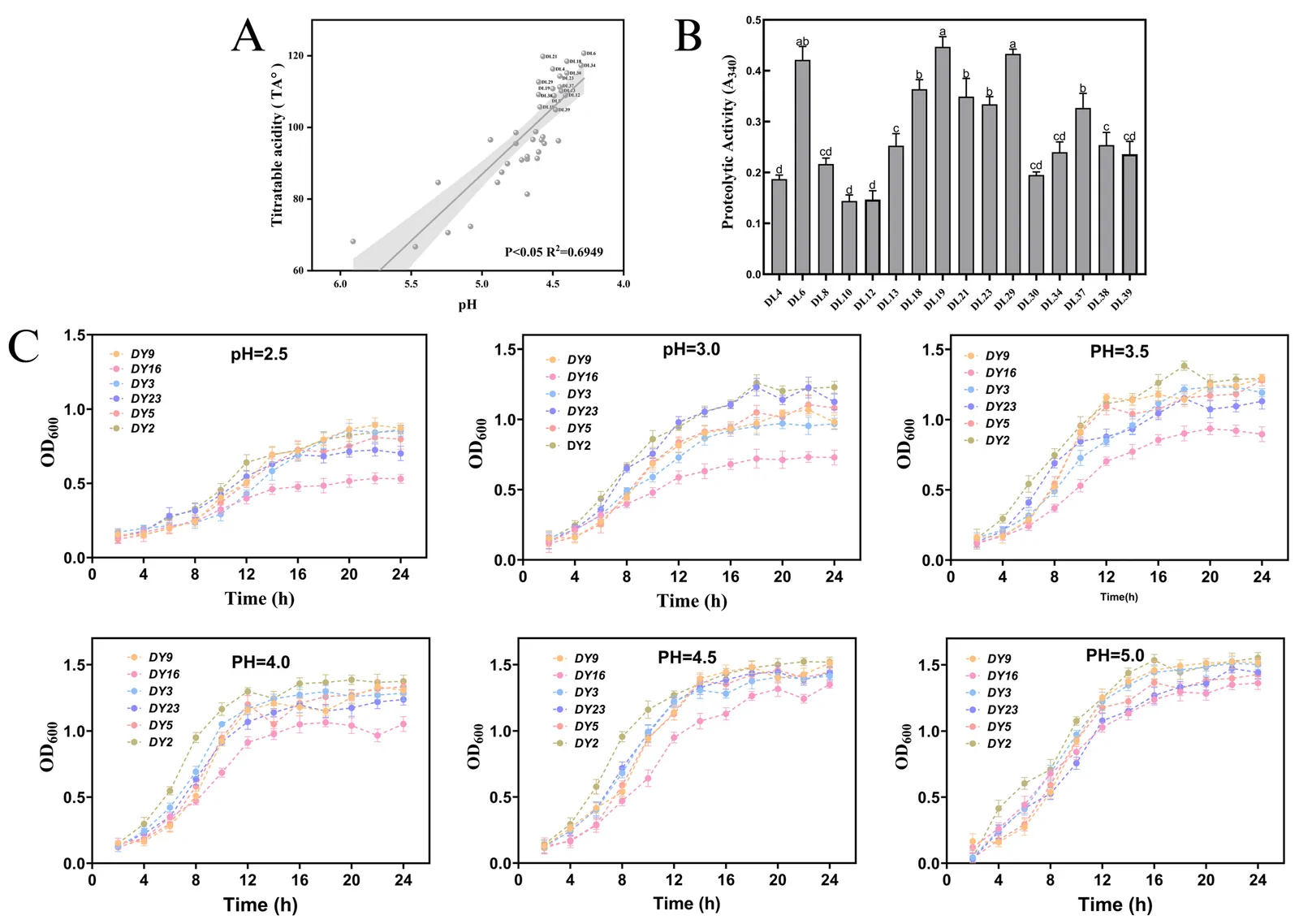
Preliminary screening of LAB and yeast isolates. (**A**) pH and TA; (**B**) proteolytic activity of LAB (Different lowercase letters (a–d) indicate significant differences among the means (*p* < 0.05). Means sharing at least one common letter are not significantly different (*p* ≥ 0.05)); and (**C**) acid tolerance of yeast (pH 2.5–5).

pH and TA are widely used indicators of the acidifying capacity of LAB in dairy systems; however, the low lactose content and strong buffering capacity of CM generally limit efficient acidification [32]. Accordingly, pH ≤ 4.6 and TA ≥ 100 °T after 24 h of fermentation were adopted as the preliminary screening criteria, consistent with thresholds established in previous studies [33,34]. Based on these criteria, 16 of the 39 strains evaluated passed the preliminary screening (Figure 2A). Among the selected strains, DL6 showed the most pronounced acidifying performance, decreasing the pH to 4.28 and increasing TA to 120.68 °T, a level exceeding that reported for several common LAB starter cultures applied in CM fermentation [18]. A significant correlation was also observed between pH and TA (R^2^ = 0.6949, *p* < 0.05), indicating that the two parameters consistently characterized the overall fermentation.

Beyond pH and TA, the proteolytic activity of the screened strains was further evaluated using the OPA method, as free amino acids and small peptides generated during fermentation directly contribute to flavor and also serve as important precursors of volatile flavor compounds, such as aldehydes, alcohols, acids, and esters, via downstream metabolic pathways [35]. OPA values varied significantly among the tested strains (Figure 2B). DL19 and DL29 exhibited the highest proteolytic activity, followed by DL6, indicating that these strains possessed both efficient acidification and strong proteolytic capacity. DL18, DL21, DL23, and DL37 displayed moderate OPA values, suggesting additional potential for flavor enhancement. By contrast, the remaining strains, despite meeting the acidification criteria, exhibited limited proteolytic activity, which may restrict subsequent flavor development and the generation of volatile flavor compounds.

### 3.3. Preliminary Screening of Yeast

The aroma and gas production characteristics of fermented milk inoculated with each of the 25 yeast isolates are summarized in Table 3. The fermented milk inoculated with DY9 and DY23 exhibited a pronounced lactic acid flavor and strong gas production, while fermented milk from DY2, DY3, DY5, and DY16 tended to have floral and ethanol flavors. Therefore, based on flavor and gas production characteristics, these six yeast strains were selected for further screening and research.

In fermented dairy systems, LAB are the primary drivers of acidification, while yeast strains are major contributors of alcohols and esters, which enrich the aroma complexity of the final product. To assess the suitability of the selected strains for CM fermentation, their acid tolerance was evaluated across a pH gradient from 2.5 to 5.0 (Figure 2C). At pH 5.0 and 4.5, the inhibitory effect of the acidic environment on cell proliferation was minimal, and all six strains maintained comparable growth. As the pH decreased to 4.0 and 3.5, acid-induced growth inhibition became more pronounced. DY2 retained rapid initial growth and high final biomass under these conditions, whereas DY16 exhibited substantially reduced growth at pH 3.0–4.0, with a final OD_600_ consistently lower than that of the other strains, indicating heightened sensitivity to acid stress. Under highly acidic conditions (pH 3.0 and 2.5), proliferation was significantly suppressed across all strains, with DY16 remaining the poorest growth performer. These results indicated that the reduced growth of DY16 within the acidic range (pH 2.5–4.0) suggested limited tolerance to progressive acidification, and this strain was therefore excluded from subsequent evaluation.

Based on the preliminary screening of LAB (pH and TA, and proteolytic activity) and yeast (gas and aroma production, and acid tolerance), seven LAB strains (DL6, DL18, DL19, DL21, DL23, DL29, and DL37) and five yeast strains (DY2, DY3, DY5, DY9, and DY23) were selected for further texture and rheological evaluation.

### 3.4. Syneresis

**Figure 3:**
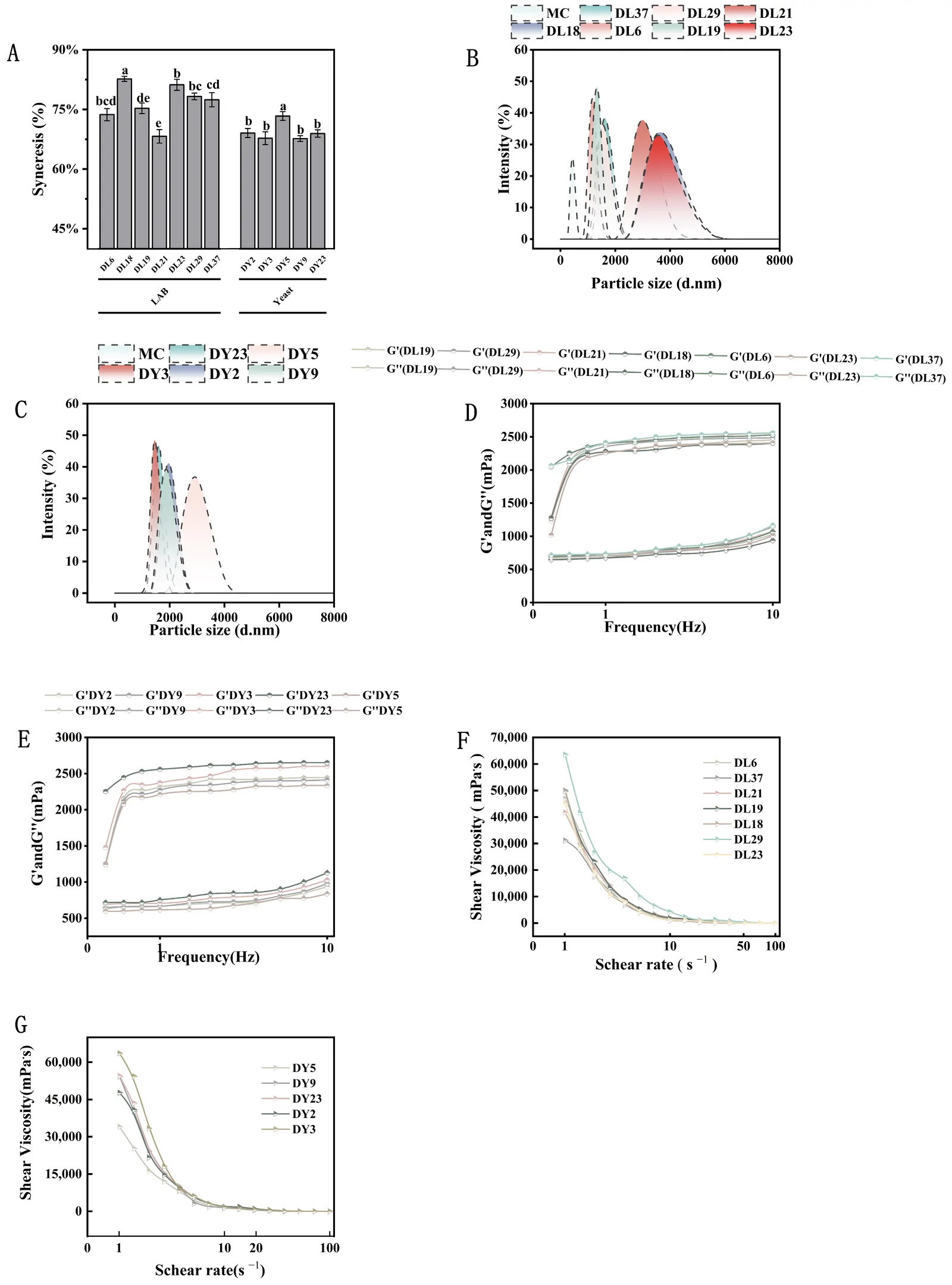
Texture and rheological properties of LAB- and yeast-fermented milk. (**A**) Syneresis (Different lowercase letters (a–e) indicate significant differences among the means (*p* < 0.05). Means sharing at least one common letter are not significantly different (*p* ≥ 0.05))**;** (**B**) particle size distribution of LAB; (**C**) particle size distribution of yeast; (**D**) changes in the apparent viscosity of LAB; (**E**) changes in the apparent viscosity of yeast; (**F**) viscoelastic properties of LAB; and (**G**) viscoelastic properties of yeast.

Syneresis is an important indicator of the physical stability of fermented milk, reflecting the extent of whey separation from the gel network. As shown in Figure 3A, syneresis varied significantly among the LAB strains. DL21 exhibited the lowest syneresis (68.2%), followed by DL6 (73.7%) and DL19 (75.3%), all of which were significantly lower than those of DL18 (82.67%) and DL23 (81.78%) (*p* < 0.05). Extracellular polysaccharides produced by LAB can interact with the protein matrix, filling the voids within the gel network and reducing whey separation and syneresis of fermented milk [36]. Since both DL18 and DL23 are *S. thermophilus*, their higher syneresis may be related to insufficient extracellular polysaccharide production [37]. Among the yeast-fermented samples, DY5 showed the highest syneresis (73.34%), while the remaining samples did not differ significantly from one another (*p* > 0.05), with syneresis values ranging from 67.7% to 69.1% (Figure 3A).

### 3.5. Particle Size Distribution

The particle size distributions of all fermented CM samples are shown in Figure 3B (LAB group) and Figure 3C (yeast group). Unfermented CM (MC) displayed a single, narrow peak at 420.2 nm. Following fermentation, the particle size distributions of all samples shifted toward larger sizes, indicating the formation of larger particle or aggregate structures. The PDI values of the fermented CM samples ranged from 0.165 to 0.618, indicating strain-dependent differences in the breadth of particle-size distributions (Table S3). In the LAB group, DL18, DL21, and DL23 exhibited main peak positions at 3477, 2990, and 3477 nm, with corresponding peak widths of 1712, 1472, and 1712 nm, respectively, indicating relatively broad particle-size distributions. DL18 and DL23 also exhibited relatively higher PDI values of 0.599 ± 0.035 and 0.618 ± 0.042, respectively, further supporting their broader particle-size distributions. In contrast, DL6 and DL19 showed smaller main peak positions of 1209 and 1406 nm, with peak widths of 367 and 197 nm, respectively, indicating relatively narrower particle-size distributions. The relatively large and broad particle-size distributions observed for DL18 and DL23 may be associated with their higher syneresis. This observation is consistent with Torres et al. [38], who reported that increased aggregate size within the protein matrix can be associated with reduced water retention. Excessive aggregation of milk protein particles may contribute to a coarser gel structure and facilitate whey separation. It is worth noting that DL21 exhibited both a relatively large particle size and low syneresis, which may be attributable to interactions among aggregated protein particles and casein micelles that promote a more interconnected gel network and enhanced water retention [39]. In the yeast group, DY5 (*Saccharomyces cerevisiae*) showed a relatively large main peak position (2990 nm) and peak width (1266 nm), whereas DY3 exhibited a markedly smaller main peak position (1460 nm) and narrower peak width (426 nm). DY5 had a higher PDI value (0.399 ± 0.047) than DY3 (0.171 ± 0.016), further supporting the difference in particle-size distribution breadth between these two samples. Previous studies have suggested that yeast growth and yeast–protein interactions can affect the continuity of the developing milk gel network [40]. Such strain-specific effects may contribute to the relatively high syneresis observed for DY5 and may influence the structural stability of fermented CM gels.

### 3.6. Apparent Viscosity and Viscoelastic Properties

The flow behavior of all fermented CM samples was characterized by a continuous decrease in apparent viscosity with increasing shear rate from 0.01 to 100 s^−1^ (Figure 3D,E), consistent with typical shear-thinning (pseudoplastic) behavior widely reported in fermented dairy products. This rheological response arises from the progressive disruption of protein–protein and protein–polysaccharide interactions under applied shear stress, leading to partial disintegration of the gel network and orientation of macromolecular chains along the flow direction [20]. At a shear rate of 10 s^−1^, substantial differences in apparent viscosity were observed among the LAB-fermented samples. DL29 exhibited the highest apparent viscosity (4199.24 mPa·s), indicative of a dense gel network with strong resistance to deformation. DL21 (1955.72 mPa·s) and DL19 (1809.72 mPa·s) also retained elevated viscosities, whereas DL18 (888.29 mPa·s), DL23 (1054.23 mPa·s), and DL37 (1027.82 mPa·s) showed markedly lower values, suggesting weaker structural integrity. In the yeast group, DY2 showed the highest viscosity (1809.04 mPa·s), while DY5 showed the lowest (1117.88 mPa·s), consistent with the higher syneresis and larger particle size observed for this strain.

Oscillation frequency scanning further confirmed the viscoelastic characteristics of the fermented CM gel, and the results are shown in Figure 3F (LAB group) and Figure 3G (yeast group). Across the entire frequency range examined (0.1–10 Hz), G′ exceeded G″ for all samples, and both moduli increased with frequency (G′ > G″), consistent with weak gel behavior widely reported in fermented dairy products [41]. In the LAB group, G′ and G″ values at 1 Hz ranged from 2257.89 to 2410.27 mPa and 672.99 to 732.25 mPa, respectively, with limited variation among strains. This relatively low gel stiffness may be attributed to the unique compositional and structural characteristics of CM. The larger casein micelle size and low *κ*-casein content collectively lower the spatial density of inter-micellar contact points and reactive aggregation sites during acidification. Furthermore, the absence of *β*-lactoglobulin limits the structural contribution of the whey protein fraction to the network [42,43]. In the yeast group, G′ values at 1 Hz ranged from 2210.75 to 2559.91 mPa, and G″ ranged from 615.97 to 752.46 mPa. DY5 exhibited the lowest G′ (2210.75 mPa) and G″ (615.97 mPa), indicating the weakest elastic response among the yeast-fermented samples, which is consistent with its high syneresis and large particle size reported above. DY23 showed the highest G′ (2559.91 mPa) and G″ (752.46 mPa), suggesting greater gel elasticity and network rigidity.

### 3.7. E-Nose

**Figure 4:**
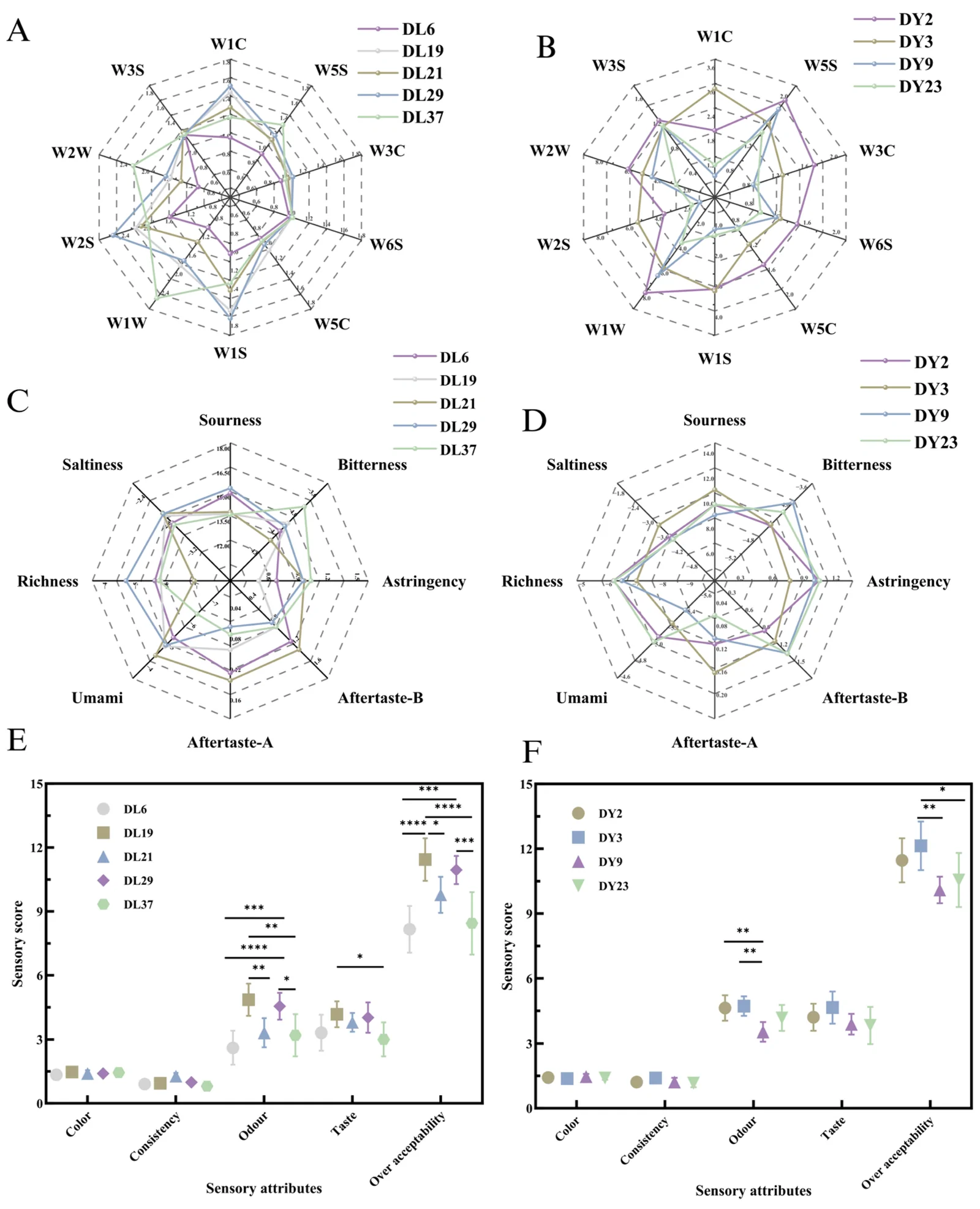
Flavor characteristics of fermented CM with different single-strain cultures. (**A**) E-nose radar plots of LAB; (**B**) E-nose radar plots of yeast; (**C**) E-tongue radar plot of LAB; (**D**) E-tongue radar plot of yeast; (**E**,**F**) sensory scores of the LAB group (**E**) and yeast group (**F**) for color, consistency, odor, taste, and overall acceptability. Significant differences between samples are denoted by asterisks (* *p* < 0.05, ** *p* < 0.01, *** *p* < 0.001, **** *p* < 0.0001).

Based on the combined assessment of textural parameters (syneresis, particle size, apparent viscosity, and viscoelastic properties), five LAB strains (DL6, DL19, DL21, DL29, and DL37) and four yeast strains (DY2, DY3, DY9, and DY23) were selected for flavor evaluation. Four of the ten MOS sensors were selected as sensory-relevant indicators for interpretation of fermented CM aroma: W1C (aromatic compounds), W2S (alcohols, aldehydes, and ketones), and W1W and W2W (sulfur-containing compounds). The response ranges of these sensors are closely related to ideal fermentation-related aromas as well as to potential off-odor compounds and therefore receive special attention. For the LAB group (Figure 4A), DL19 and DL29 elicited the highest responses on W1C and W2S sensors, indicating stronger sensor responses associated with fruity, creamy, and fermented characteristics [44]. DL37 produced pronounced responses on the W1W and W2W sensors, indicating stronger responses on sensors associated with sulfur-containing volatiles, at high concentrations, may contribute to undesirable sulfurous off-notes. By contrast, DL6 displayed uniformly low responses across all sensors; despite its strong acidifying capacity, its aroma contribution was limited. This may be partly explained by a high glycolytic flux and efficient lactate dehydrogenase (LDH)-mediated conversion of pyruvate to lactate, which diverts carbon flux away from the synthesis of aroma-active compounds such as aldehydes, ketones, and esters [45]. Regarding yeast samples (Figure 4B), DY3 exhibited the highest responses on the W1C and W2S sensors, indicating a robust capability to generate alcohols, esters, and aroma-active volatiles that enrich fruity and fermented notes. In contrast, DY9 showed limited responses on these channels but intense signals on sulfur-sensitive sensors, suggesting a restricted flavor profile accompanied by pungent off-notes that could compromise consumer acceptance.

### 3.8. E-Tongue

E-tongue analysis revealed strain-specific modulation of CM taste, with the most evident variations observed in sourness, bitterness, astringency, umami, and saltiness. These attributes represent the primary flavor attributes directly related to the sensory characteristics of fermented dairy products [46]. Among the LAB samples (Figure 4C), all strains enhanced the sourness response relative to MC, with DL6 and DL29 showing the strongest increases, consistent with their high acidifying capacity and indicative of greater accumulation of lactic acid and other organic acids during fermentation. By contrast, DL19 and DL21 markedly reduced bitterness and astringency. This reduction is likely related to the activity of exopeptidases, which further break down the high concentrations of hydrophobic peptides derived from β-casein hydrolysis into free amino acids. This process disrupts the hydrophobic structures essential for receptor binding, preventing them from contacting TAS2R receptors and thereby reducing bitterness [47,48,49]. Conversely, an imbalance favoring endopeptidase activity can lead to the accumulation of intact bitter peptides, explaining the relatively high residual bitterness and astringency of DL23 [48]. The pronounced decline in umami and increase in bitterness of DL37 further indicated a less harmonious taste profile, in agreement with its lower sensory acceptability and elevated sulfur-sensor response in the E-nose analysis. A reduced saltiness response was observed for all fermented samples relative to MC, with DL29 and DL21 showing the greatest decreases. This change is more plausibly explained by perceptual taste interactions, in which the high concentrations of organic acids inhibit or reduce the perception of saltiness [50]. Among the yeast samples (Figure 4D), DY3 displayed the most balanced taste profile, with well-coordinated sourness, umami, and richness, and the greatest reduction in saltiness. DY2 and DY23 retained moderate umami intensity and taste complexity but were less balanced overall than DY3, whereas DY9 showed reduced umami and richness alongside heightened bitterness and astringency, suggesting the formation of metabolites detrimental to taste balance and a correspondingly lower sensory acceptability.

### 3.9. Sensory Evaluation

The sensory properties of the fermented CM samples were evaluated using a weighted scoring model. For the LAB-fermented samples (Figure 4E), color and consistency did not differ significantly among strains (*p* > 0.05). In contrast, odor scores varied significantly, with DL19 and DL29 rated highest and DL6 rated lowest, in agreement with the E-nose results. For taste, a significant difference was observed between DL19 and DL37 (*p* < 0.05), the latter receiving the lowest score. Overall acceptability followed a similar trend: DL19 achieved the highest score, significantly exceeding those of DL6 (*p* < 0.0001) and DL37 (*p* < 0.001), followed by DL29 and DL21, whereas DL6 and DL37 were rated lowest. For the yeast-fermented samples (Figure 4F), no significant differences were detected in color, consistency, or taste (*p* > 0.05), whereas odor and overall acceptability differed significantly among strains. DY3 obtained the highest overall sensory score, significantly higher than that of DY9 (*p* < 0.05) but comparable to that of DY2 (*p* = 0.6187), while DY9 was rated lowest in overall acceptability.

The integrated E-nose, E-tongue, and sensory analyses indicated that DL6 (weak aroma contribution) and DL37 (residual bitterness, astringency, and sulfurous notes) among the LAB strains, and DY9 (low umami and richness with pronounced bitterness) among the yeast strains, exhibited inferior flavor performance and were excluded. Accordingly, DL19, DL21, and DL29, together with DY2, DY3, and DY23, were selected for subsequent volatile flavor profiling by HS-SPME-GC-MS.

### 3.10. Volatile Flavor Compounds

**Figure 5:**
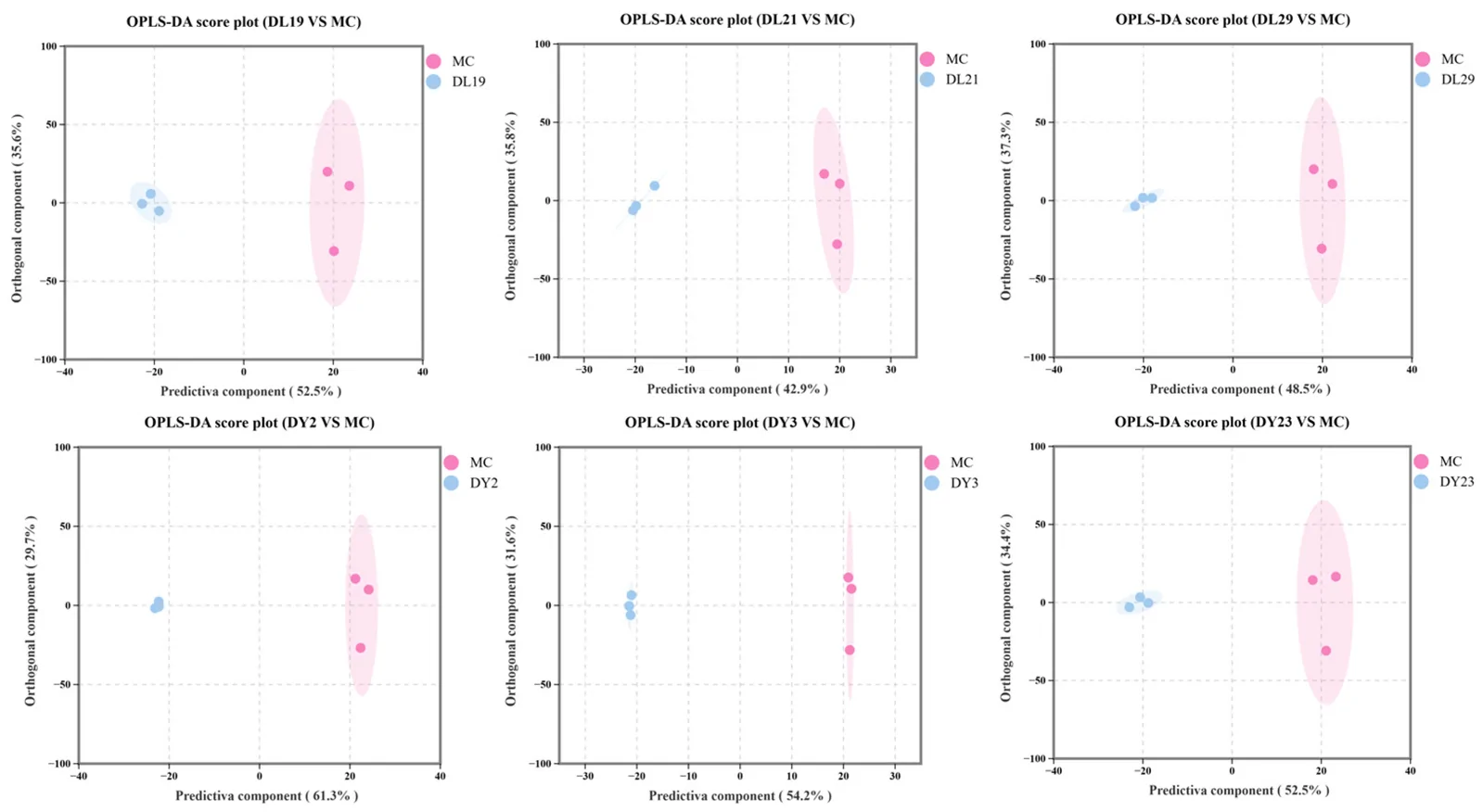
OPLS-DA score plots of each fermented sample compared with MC.

To characterize the effects of fermentation on volatile compound formation, the volatile metabolite profiles of MC and the six selected fermented CM samples were analyzed by HS-SPME-GC-MS, and a total of 1456 volatile metabolites were identified. Orthogonal partial least squares discriminant analysis (OPLS-DA) revealed a clear separation between the control and fermented groups along the predictive component, confirming that fermentation substantially reshaped the volatile landscape (Figure 5). Permutation tests verified model robustness and no apparent overfitting (Figure S3).

**Figure 6:**
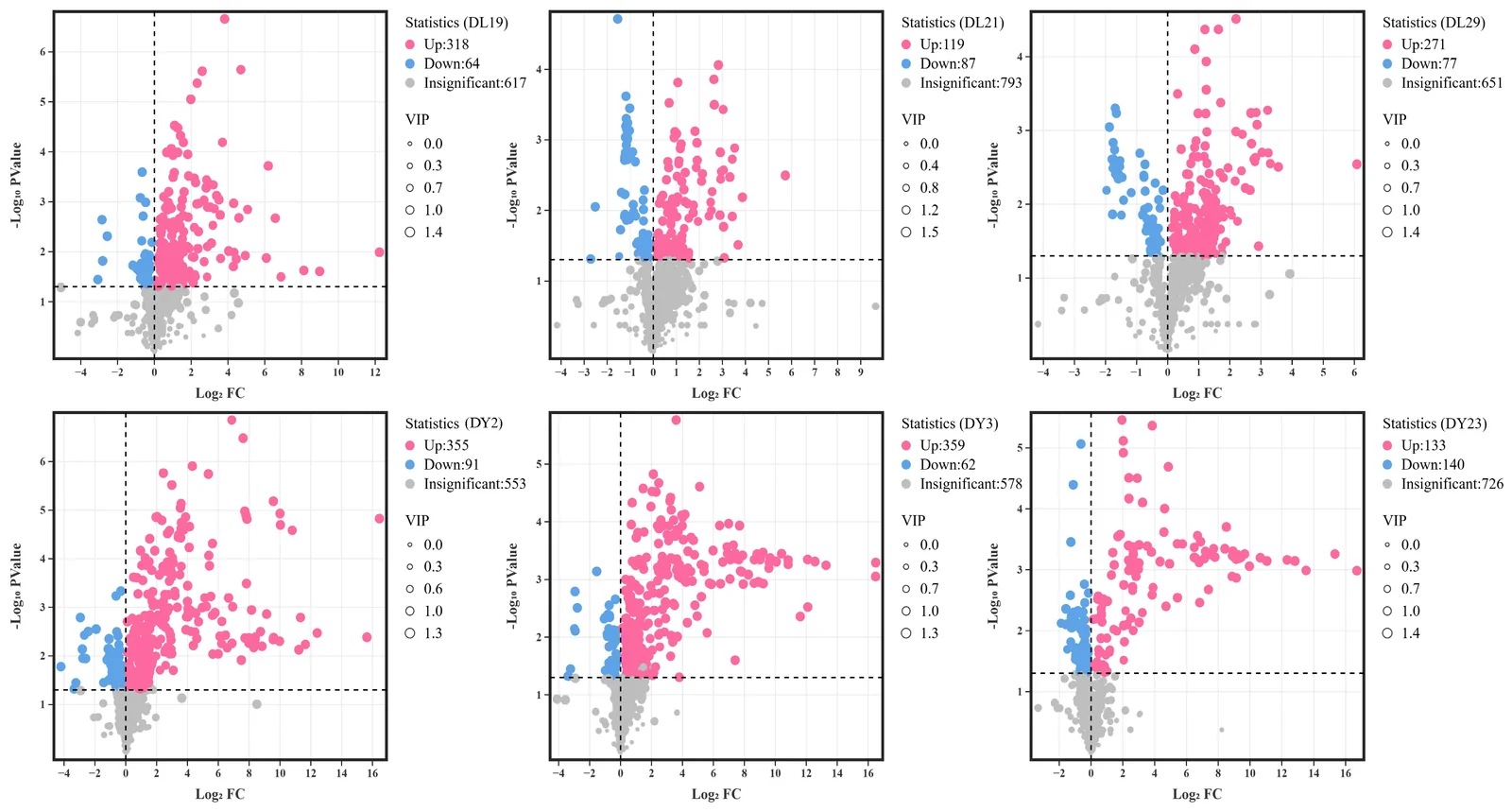
Volcano plots illustrate significantly up- and down-regulated metabolites.

Differential volatile metabolites were identified using combined multivariate and univariate thresholds (VIP ≥ 1, *p* < 0.05, and |log_2_FC| > 1) and visualized using volcano plots (Figure 6). DY3 and DY2 displayed the highest numbers of upregulated metabolites (359 and 355), followed by DL19 (318) and DL29 (271), whereas DL21 and DY23 showed fewer changes (119 and 133). Conversely, DY23 exhibited the highest number of downregulated metabolites (140), followed by DY2 (91), DL21 (87), and DL29 (77), while DL19 and DY3 showed the fewest (64 and 62, respectively) (Figure 6). Notably, DY3 and DL19 were characterized by extensive upregulation accompanied by limited downregulation, indicating a strong capacity to generate and accumulate volatile compounds. Although DY23 was also clearly distinguished from MC, its downregulated metabolites outnumbered the upregulated ones, suggesting that this strain transforms or consumes existing volatile substrates rather than accumulating new products.

**Figure 7:**
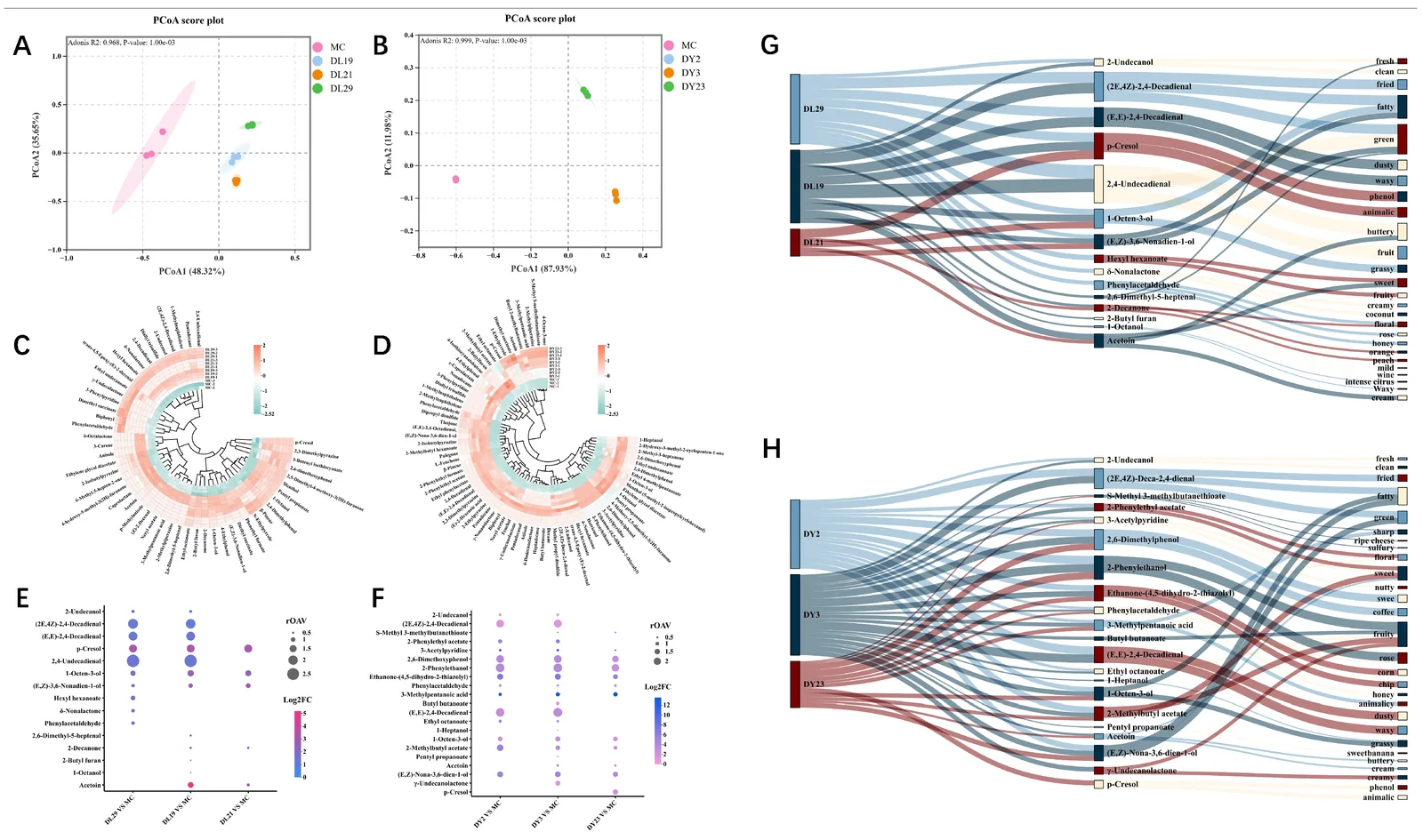
Volatile compound profiles of the different fermented CM samples. (**A**,**B**) PCoA score plots of the LAB group (**A**) and yeast group (**B**); (**C**,**D**) circular heatmaps of the differential volatile compounds, showing relative abundance patterns and compositional changes in the LAB group (**C**) and yeast group (**D**); (**E**,**F**) bubble plots showing the rOAV and log_2_FC of the key flavor compounds of the LAB (**E**) and yeast group (**F**); (**G**,**H**) Sankey diagrams linking the differential volatile compounds to their reported odor descriptors of the LAB group (**G**) and yeast group (**H**).

Because statistical significance does not necessarily translate into sensory relevance, the differential volatile metabolites were further refined according to odor descriptors reported in the literature and flavor databases, excluding compounds without defined odor characteristics. On this basis, 50 volatile flavor compounds were retained for the LAB group (Figure 7C), comprising nine esters, six ketones, five aldehydes, four alcohols, seven benzene derivatives, two lactones, six heterocyclic compounds, three hydrocarbons, three terpenes, one organic acid, two ethers, and two other compounds. Among these LAB isolates, DL19 harbored the greatest number (43), followed by DL29 (32) and DL21 (27). For the yeast group, 74 volatile flavor compounds were recovered (Figure 7D), including 14 esters, eight ketones, six aldehydes, seven alcohols, eight benzene derivatives, four lactones, seven heterocyclic compounds, five hydrocarbons, six terpenes, two organic acids, four ethers, and three other compounds, where DY3 and DY2 retained 67 and 61 compounds, respectively, and DY23 retained 34. Collectively, yeast fermentation may generate a wider spectrum of volatile compounds than LAB fermentation, particularly regarding esters, alcohols, and lactones, potentially promoting a more diverse flavor profile. However, this difference cannot be entirely attributed to the type of strain.

Principal coordinate analysis (PCoA) was subsequently performed to evaluate these odor-active variations. Within the LAB (Figure 7A), the first two coordinates explained 49.87% and 31.40% of the total variance, whereas they accounted for 87.65% and 12.22% in the yeast (Figure 7B). Biological replicates clustered closely within each strain, indicating good within-strain consistency, while distinct separation among samples demonstrated differences in volatile profiles among the fermented CM samples. Notably, DY2 and DY3 partially overlapped along both coordinates, indicating similar flavor-forming metabolic behavior that may stem from their close taxonomic relationship within the same Kluyveromyces lineage. Even so, these results were consistent with strain-dependent differences in the volatile composition of fermented CM.

### 3.11. rOAV Analysis

rOAV were calculated to identify the volatile compounds most relevant to aroma perception, with rOAV ≥ 1 indicating a meaningful contribution to the overall flavor profile. On this basis, 12 and 10 key flavor-active compounds were identified in DL19 and DL29, respectively, compared with only 5 in DL21 (Figure 7E). Concurrently, DL19 and DL29 exhibited remarkably higher total rOAVs (677.15 and 702.17) than DL21 (83.62) (Table 4), indicating a richer odor-active profile under the tested fermentation conditions. The aroma profiles of DL19 and DL29 were dominated by aldehydes, particularly 2,4-undecadienal and (2E,4Z)-2,4-decadienal. These compounds primarily impart desirable green and buttery notes, while their characteristic greasy and waxy nuances collectively establish rich, full-bodied fatty notes (Figure 7G). This distinctive character indicates that the high aldehyde-driven rOAVs significantly enhance the overall aroma complexity. Among all samples, 2,4-undecadienal and *δ*-nonalactone were exclusively detected in the LAB-fermented samples, suggesting that these compounds may serve as characteristic aroma markers of the LAB strains examined. This observation is consistent with the findings of Duo et al. [51], who reported a significant association between LAB and the accumulation of 2,4-undecadienal and *δ*-nonalactone. In DL19, acetoin served as the primary source of the buttery and milky notes, exhibiting an rOAV 4.2-fold higher than that of DL21 [52]. Although acetoin was completely absent in DL29, this strain uniquely synthesized a combination of floral, fruity, and lactonic compounds among the tested LAB, including phenylacetaldehyde (honey, floral), hexyl hexanoate (sweet, fruity), and *δ*-nonalactone (creamy, milky). The simultaneous presentation of these sweet, floral, and coconut-like nuances provides a sensory masking effect, effectively offsetting the greasy and waxy undertones of the high rOAV aldehyde and establishing a well-harmonized flavor profile for DL29. Conversely, the sparse volatile profile of DL21 was dominated by p-cresol, 1-octen-3-ol, and (3E,6Z)- 3,6-nonadien-1-ol, whose prominent animalic, phenolic, and mushroom off-odors generated a highly unharmonious volatile profile, rendering DL21 unsuitable as a flavor-enhancing strain.

Among the yeast strains, DY3 produced the most complex volatile profile, with 20 key flavor compounds (rOAV ≥ 1), compared with 14 and 12 in DY2 and DY23, respectively (Figure 7F). The total rOAVs of DY2 (929.42) and DY3 (983.65) were approximately three times that of DY23 (297.67), indicating a less diverse odor-active profile in DY23. The differential flavor compounds in the yeast group were dominated by alcohols and esters. 2-phenylethanol, an Ehrlich-pathway-derived alcohol, was the single strongest contributor across all yeast strains, with rOAVs of 245.15 (DY2), 242.28 (DY3), and 114.17 (DY23) (Table 4). Interestingly, the study by Bilal et al. [6] also found that 2-phenylethanol contributes significantly to the flavor of shubat. Its high accumulation is likely attributable to the reduction in phenylacetaldehyde by NAD(P)H-dependent alcohol dehydrogenases and aldehyde reductases, yielding characteristic rose- and honey-like notes [53,54]; this may partly account for the much lower rOAV of phenylacetaldehyde relative to 2-phenylethanol. The most pronounced differences were observed in the ester fraction (Table 4). DY2 accumulated an exceptionally high level of 2-methylbutyl acetate, imparting a ripe banana-like note, followed by DY3 (16.90) and DY23 (9.38). In contrast, butyl butanoate (peach, tropical fruit), pentyl propanoate (apricot), and *γ*-undecalactone (peach, creamy) were detected exclusively in DY3, further enriching its fruity character. Although the high rOAVs of (E, E)-2,4-decadienal in DY2 and DY3 indicated potentially strong fatty, oily, and oxidation-related notes, their sensory expression may have been attenuated by the abundant fruity esters and 2-phenylethanol, which can enhance pleasant fruity and floral attributes and suppress the perception of certain off-odors through non-additive aroma interactions [55,56]. This apparent advantage was less evident in the LAB-fermented samples, which contained lower ester levels. However, this difference cannot be attributed solely to microbial type, as LAB and yeasts were fermented under different temperatures, inoculation levels, and initial cell concentrations. S-methyl 3-methylbutanethioate, a sulfur-bearing compound, was detected in both DY3 and DY23 (Figure 7H). Interestingly, only one sulfur-containing compound was detected among the key flavor compounds, which appears inconsistent with the strong responses of the W1W and W2W sensors in the E-nose analysis. Yuan et al. [57] noted that this may be due to the high sensitivity of E-nose sensors to sulfur-containing compounds, as well as synergistic effects among various volatile compounds that enhance the overall sulfur odor response signal. Notably, DY23 lacked most of the characteristic esters and lactones found in DY2 and DY3, yet contained the highest level of 3-methylpentanoic acid, a compound associated with sweaty and cheesy off-odors (Table 4). Together, these features likely explain the lower sensory acceptability and weaker flavor complexity of DY23.

Based on the comprehensive rOAV profiles, DL19 and DL29 were selected as the LAB candidates due to their flavor traits—DL19 provided a powerful buttery foundation via acetoin, whereas DL29 offered a multi-layered floral and lactonic harmony. Within the yeast group, although DY2 and DY3 were identified as the same species, DY3 displayed a superior capacity to synthesize various esters and lactones, thereby uniquely imparting a broader spectrum of fruity notes. Consequently, DL19, DL29, and DY3 were selected as candidates for further evaluation under their respective fermentation conditions.

### 3.12. Genome Analysis and Preliminary Safety Assessment

Whole-genome sequencing (WGS) analysis was performed to further assess safety-related genomic characteristics of the three selected strains [58] (Table S4). CARD-based screening of the LAB yielded 70 and 79 homologous matches for DL19 and DL29, respectively, but none showed ≥80% amino acid identity [59]. Most matches corresponded to conserved chromosomal proteins, transporters, or mutation-dependent resistance targets rather than clear acquired antimicrobial-resistance determinants. VFDB analysis identified three homologs in DL19 (*LisR*, *Tuf*, and *HasC*) and six in DL29 (*LisR*, *Eno*, *ClpP*, *TufA*, *HasC*, and *Bsh*). These predominantly represented conserved regulatory, metabolic, stress-response, or housekeeping proteins, and no clear canonical toxin- or invasion-associated determinant was evident in the supplied VFDB results [60]. Among the safety-related genomic features examined, an ornithine decarboxylase homolog was detected in DL19, whereas no obvious histidine, tyrosine, or ornithine decarboxylase was identified in DL29 under the present annotation framework.

For the yeast strain DY3, screening against DFVF and PHI-base using criteria of identity ≥80% and aligned length ≥100 aa generated numerous homologous annotations, but these were dominated by conserved cellular and stress-response proteins, including *HOG1*, *CDC42*, *TPK2*, and related signaling or metabolic proteins [61]. No clear classical Candida-associated pathogenicity markers, such as *ECE1/candidalysin*, *HWP1*, *ALS1/ALS3*, or the typical *SAP* virulence repertoire, were evident in the supplied annotation outputs. Because PHI-base and DFVF contain genes whose homologs may contribute to virulence in pathogenic fungi, their presence in *K. marxianus* was not interpreted as direct evidence of pathogenicity. Overall, the genomic analyses did not reveal obvious major classical pathogenicity determinants in DL19, DL29, or DY3. However, the ornithine-decarboxylase-related signal in DL19 warrants targeted phenotypic confirmation.

## 4. Conclusions

Candidate strains for CM fermentation were selected using a multi-tiered framework integrating functional screening, textural characterization, and standardized flavor evaluation. From 198 LAB and 120 yeast isolates obtained from traditionally fermented CM, seven LAB and five yeast strains were selected for advanced evaluation, and six strains were further subjected to HS-SPME-GC-MS and rOAV analysis. Fermentation markedly altered the volatile profile of CM in a strain-dependent manner. In addition, WGS analysis was used to further elucidate the safety profile of the candidate strains. Overall, DL19 (*Lacticaseibacillus paracasei*), DL29 (*Lactiplantibacillus plantarum*), and DY3 (*Kluyveromyces marxianus*) showed favorable fermentation properties and clear flavor-enhancing potential, making them promising candidates for starter or adjunct cultures. The standardized multi-criteria framework used in this study provides a consistent basis for comparing strains and selecting candidates for controlled flavor-oriented fermentation of CM. Further studies should focus on LAB–yeast co-fermentation, inoculation ratios, and process optimization to improve flavor consistency and fermentation performance. Multi-omics approaches may further clarify the relationships between microbial composition, metabolic functions, and flavor formation. In addition, safety phenotypic validation of the strains and evaluation of potential probiotic properties will be necessary before practical application, together with improved microbiological standardization of fermented CM production.

## Figures and Tables

**Table 1:** Basic composition parameters of fresh CM.

Parameter	Unit	Content (Mean ± SD)
Protein	g/100 g	3.78 ± 0.03
Fat	g/100 g	5.02 ± 0.07
Lactose	g/100 g	4.90 ± 0.00
Total soluble solids	°Brix	11.70 ± 0.10

**Table 2:** Sensory attributes, weighting factors (Fi), and score ranges used in the sensory evaluation of fermented CM.

Attribute	Fi	Highest Score	Lowest Score
color	0.4	5	0
consistency	0.4	5	0
odor	1.2	5	0
taste	1.2	5	0

**Table 3:** Aroma characteristics and gas production of fermented CM produced by the 25 yeast isolates.

Aroma Characteristics	Gas Production	Strain
Lactic acid flavor	Strong	DY9, DY23
Ethanol flavor	Moderate	DY2, DY3, DY5, DY16
No obvious characteristic flavor	Weak	DY4, DY6, DY7, DY8, DY11, DY13, DY14, DY17, DY18, DY20, DY21, DY22, DY24
Off flavor	None	DY1, DY10, DY12, DY15, DY19, DY25

**Table 4:** rOAV Values for various volatile compounds in fermented milk samples.

Category	Aroma Substance	Aroma Threshold (μg·mL^−1^)	Aroma Description	rOAVs					
				DL19	DL21	DL29	DY2	DY3	DY23
Alcohol	2-Undecanol	0.0086	fresh, waxy, clean, cloth, cotton, sarsaparilla	3.68 ± 0.47 ^a^	-	4.13 ± 0.26 ^a^	4.37 ± 0.34 ^a^	3.97 ± 0.92 ^a^	-
	1-Octen-3-ol	0.001	fatty, fruity, grassy, mushroom, perfumy, sweet	21.53 ± 0.52 ^a^	17.52 ± 2.12 ^b^	12.57 ± 0.78 ^c^	18.38 ± 0.63 ^b^	22.89 ± 0.38 ^a^	15.99 ± 2.10 ^b^
	(3E,6Z)-3,6-Nonadien-1-ol	0.003	fatty, green, cucumber, green pepper, fruity, watermelon	8.45 ± 0.85 ^c^	10.03 ± 0.44 ^c^	4.83 ± 0.51 ^c^	40.98 ± 0.88 ^a^	35.74 ± 6.29 ^a^	24.42 ± 0.26 ^b^
	1-Octanol	0.022	intense citrus, rose	1.10 ± 0.06 ^a^	-	-	-	-	-
	2-Phenylethanol	0.14	fruity, rose, sweet, apple	-	-	-	245.15 ± 1.61 ^a^	242.28 ± 5.45 ^a^	114.17 ± 4.83 ^b^
	1-Heptanol	0.003	grassy	-	-	-	-	1.17 ± 0.14 ^a^	-
Aldehydes	(2E,4Z)-2,4-Decadienal	0.00007	fried, fatty, geranium, green, waxy	108.55 ± 13.73 ^a^	-	124.77 ± 9.24 ^a^	135.51 ± 12.05 ^a^	141.12 ± 35.44 ^a^	-
	(E, E)-2,4-Decadienal	0.00007	dusty, waxy, oily, soapy	75.74 ± 11.12 ^c^	-	73.95 ± 6.84 ^c^	223.50 ± 8.17 ^b^	281.11 ± 12.96 ^a^	-
	Phenylacetaldehyde	0.0063	floral, honey, rose, cherry	-	-	5.26 ± 0.15 ^a^	4.05 ± 0.12 ^b^	3.10 ± 0.32 ^c^	3.50 ± 0.35 ^b^
	2,6-Dimethyl-5-heptenal	0.016	fresh, ozonous, melon, fresh air, sweet, green	2.01 ± 0.08 ^a^	-	-	-	-	-
	2,4-Undecadienal	0.00001	green, buttery, spicy, baked, fruity, fatty, aldehydic, chicken	389.39 ± 75.28 ^a^	-	413.63 ± 33.00 ^a^	-	-	-
Benzene and substituted derivatives	p-Cresol	0.00024	phenol, narcissus, animalic, mimosa	46.95 ± 1.92 ^a^	49.57 ± 2.37 ^a^	47.93 ± 0.55 ^a^	-	-	27.37 ± 1.37 ^b^
	2,6-Dimethylphenol	0.4	sweet, medicinal, phenol, root, coffee	-	-	-	128.52 ± 0.79 ^a^	126.74 ± 2.82 ^a^	63.25 ± 2.51 ^b^
Esters	Hexyl hexanoate	6.4	sweet, fruity	-	-	9.62 ± 0.19 ^a^	-	-	-
	S-Methyl 3-methylbutanethioate	0.1	sharp, ripe cheese, sulfury, acrid, fermented, tomato, mushroom	-	-	-	-	1.10 ± 0.02 ^b^	1.35 ± 0.08 ^a^
	2-Phenylethyl acetate	0.24959	floral, rose, sweet, honey, fruity, tropical	-	-	-	11.69 ± 0.82 ^a^	11.97 ± 0.62 ^a^	-
	Butyl butanoate	0.028	fruity, banana, pineapple, green, cherry, tropical fruit, ripe fruit, juicy fruit	-	-	-	-	7.01 ± 0.15 ^a^	-
	Ethyl octanoate	0.04	fruity, banana	-	-	-	5.09 ± 0.18 ^a^	3.20 ± 0.19 ^b^	-
	2-Methylbutyl acetate	0.005	overripe, fruity, sweet, banana, juicy, fruity	-	-	-	57.89 ± 9.25 ^a^	16.90 ± 1.66 ^b^	9.38 ± 0.69 ^b^
	Pentyl propanoate	0.085	sweet, fruity, apricot, pineapple, tropical, banana	-	-	-	-	1.00 ± 0.16 ^a^	-
Furan	2-Butylfuran	0.005	mild, fruity, wine, sweet, spicy	1.22 ± 0.02 ^a^	-	-	-	-	-
Ketones	2-Decanone	0.06	orange, floral, fatty, peach	2.43 ± 0.17 ^a^	2.69 ± 0.17 ^a^	-	-	-	-
	Acetoin	0.011	sweet, buttery, creamy, dairy, milky, fatty	16.10 ± 0.24 ^a^	3.81 ± 0.25 ^b^	-	-	3.81 ± 0.19 ^b^	3.32 ± 0.98 ^b^
	3-Acetylpyridine	0.5	sweet, nutty, dry, hawthorn, phenol, woody, popcorn	-	-	-	3.79 ± 0.03 ^a^	3.76 ± 0.08 ^a^	1.70 ± 0.08 ^b^
	Ethanone-(4,5-dihydro-2-thiazolyl)	0.001	corn, chip, taco, potato, toasted, bread, nutty	-	-	-	43.83 ± 0.55 ^a^	43.36 ± 1.24 ^a^	19.46 ± 1.26 ^b^
Lactones	δ-Nonalactone	2.6	coconut, creamy, sweet, milky, coumarin	-	-	5.48 ± 0.40 ^a^	-	-	-
	γ-Undecalactone	0.0021	fruity, peach, creamy, fatty, lactonic, apricot, ketonic, coconut	-	-	-	-	21.67 ± 3.48 ^a^	-
Organic acids	3-Methylpentanoic acid	0.046	animalic, sharp, acid, cheese, green, fruity, sweaty	-	-	-	6.67 ± 0.62 ^c^	11.75 ± 0.24 ^b^	13.76 ± 0.92 ^a^

The aroma descriptions are sourced from the following databases: http://www.thegoodscentscompany.com, accessed on 3 October 2026; http://perflavory.com/, accessed on 3 October 2026; http://www.odour.org.uk/odour/index.html, accessed on 3 October 2026); http://foodflavorlab.cn/#/home, accessed on 3 October 2026. Different lowercase letters (a–c) indicate significant differences among the means (*p* < 0.05).

## Data Availability

The data presented in this study are available on request from the corresponding author. The data are not publicly available because they are currently being used for ongoing research.

## Supplementary Materials

The following supporting information can be downloaded at: https://www.mdpi.com/article/10.3390/foods15203610/s1, Figure S1: Phylogenetic tree of LAB based on 16S rRNA gene sequences.; Figure S2: Phylogenetic tree of yeasts based on 26S rRNA sequences; Figure S3: Permutation test plots (N = 200) for validating the OPLS-DA model; Table S1: Sampling information for fermented and fresh camel milk samples collected in Xinjiang, China; Table S2: Molecular identification and sequence characteristics of representative LAB and yeast isolates. Table S3: The polydispersity index (PDI) of LAB and yeast-fermented milk; Table S4: Genomic characteristics and safety-related features of the selected strains DL19, DL29, and DY3.
