# Supplementary material for: A Flavor-Oriented Multi-Criteria Screening Strategy Identifies Indigenous Lactic Acid Bacteria and Yeasts for Improving Fermented Camel Milk Quality
Source: Foods. 2026 Oct 10;15(20):3610. doi: 10.3390/foods15203610 (PMC13703669; doi:10.3390/foods15203610)
Supplement: Supplementary file 1 [file foods-15-03610-s001.zip › foods-4554473-supplementary.pdf]

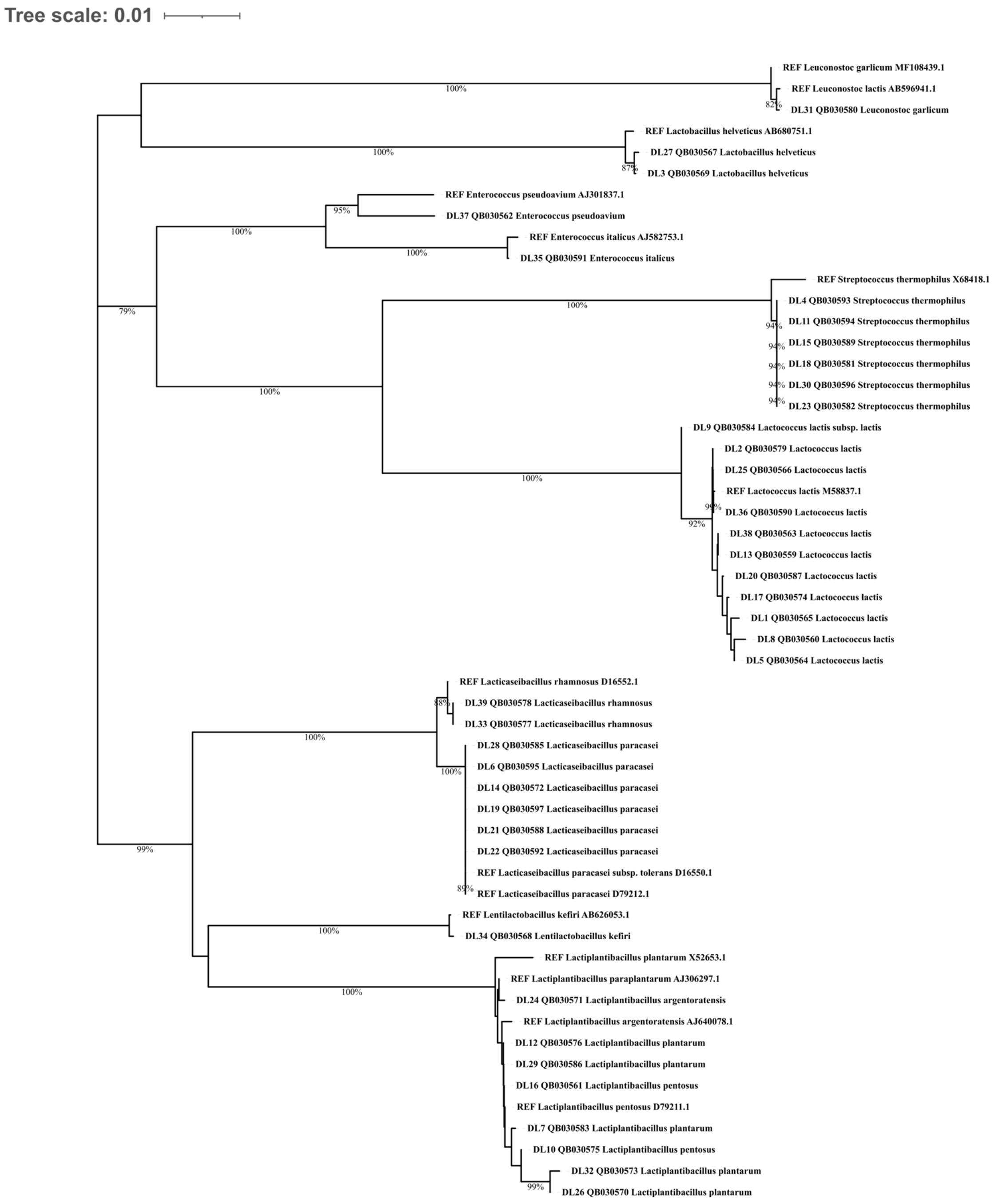


**Figure S1: Phylogenetic tree of LAB based on 16S rRNA gene sequences. Sequenceswere aligned using MUSCLE in MEGA 12, and the phylogenetic tree was reconstructed using the neighbor-joining method based on p-distances. Branch support was evaluated using 1,000 bootstrap replicates. Gaps and missing data were treated using the partial-deletion option with a site coverage cutoff of 95%, resulting in a final alignment of 1,341 nucleotide positions. Reference sequences retrieved from GenBank are labeled with their accession numbers. The scale bar represents the number of nucleotide substitutions per site.**

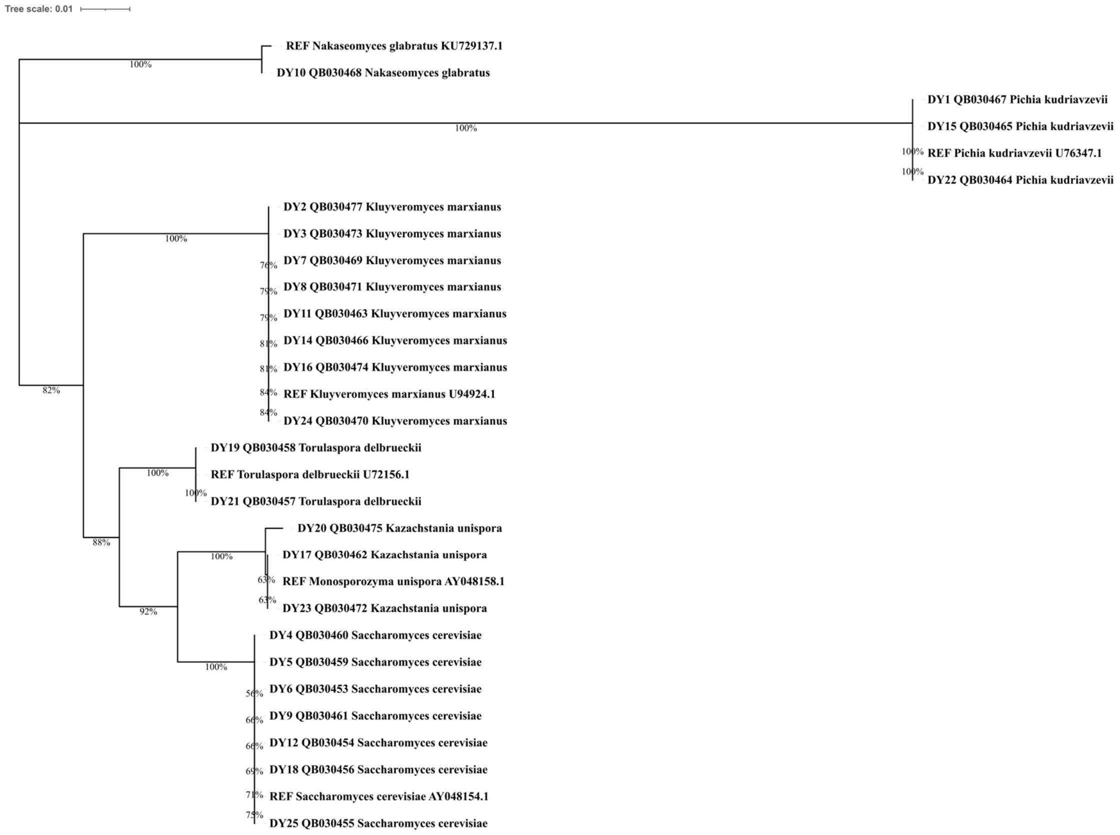


**Figure S2. Phylogenetic tree of yeasts based on the D1/D2 region of 26S rDNA sequences. Sequences were aligned using MUSCLE in MEGA 12, and the phylogenetic tree was reconstructed using the neighbor-joining method based on p-distances. Branch support was evaluated using 1,000 bootstrap replicates. Gaps and missing data were treated using the partial-deletion option with a site coverage cutoff of 95%, resulting in a final alignment of 531 nucleotide positions. Reference sequences retrieved from GenBank are labeled with their accession numbers. The scale bar represents the number of nucleotide substitutions per site.**

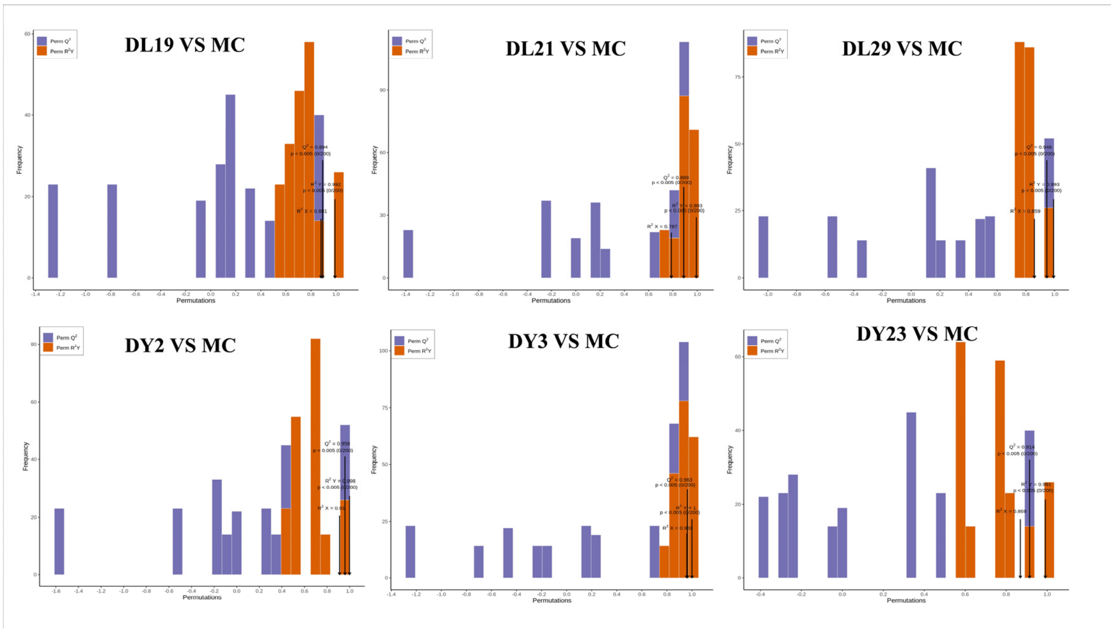


Figure S3: Permutation test plots ($N$=200) for validating the OPLS-DA model.

**Table S1. Sampling information for fermented and fresh camel milk samples collected in Xinjiang, China.**

| Sample ID | Region | Sampling site | Latitude (N) | Longitude (E) | Elevation (m) | Producer ID | pH | Sample volume |
|---|---|---|---|---|---|---|---|---|
| FCM-1 | Fuhai County, Altay | Asitaoxia Village, Qiganjiedi Township | 47°1'52" | 88°2′32″ | 564.2 | H1 | 3.57 | 60 mL |
| FCM-2 | Burqin County, Altay | Yusun Buick Ranch | 48°49'56" | 87°02′17″ | **539.3** | H2 | 3.41 | 60 mL |
| FCM-3 | Jimunai County, Altay | Bekete Village, Qaleshi Lake Township | 47°31′40″ | 86°8′34″ | **733.1** | H3 | 3.9 | 60 mL |
| FCM-4 | Huocheng County, Ili | Guozi Gou Ranch | 44°19'19" | 80°57'2" | 1004.8 | H4 | 2.01 | 60 mL |
| FCM-5 | Yining City, Ili | Nonglu Huaguoshan Community | 43°26'9" | 81°15'5″ | 889.3 | H5 | 2.5 | 60 mL |
| FCM-6 | Xinyuan County, Ili | Manhaizi Livestock Farming Cooperative, Tuergen Township | 43°31'39" | 83°25′54″ | 943.8 | H6 | 2.72 | 60 mL |
| FCM-7 | Kuche City, Aksu | The intersection of Tianshan East Road and | 41°41'34" | 82°54′13″ | 983 | H7 | 2.52 | 60 mL |

| | | | | | | | | |
|---|---|---|---|---|---|---|---|---|
| FCM-8 | Kuche City, Aksu | Changning Road in Dongcheng Street Tianshan West Road, Old City | 41°43'33" | 82°55'59" | 1002.8 | H8 | 1.96 | 60 mL |
| FCM-9 | Luntai County, Aksu | Yigenbage Village, Luntai Town | 41°45′17" | 84°15′54" | 902.9 | H9 | 3.05 | 60 mL |
| FCM-10 | Mulei County, Changji | Xiaquan Village Long-Eyebrowed Camel Breeding Base | 43°49'48" | 90°16'55" | 1219.6 | H10 | 2.44 | 60 mL |
| FCM-11 | Mulei County, Changji | - | 43°55′48″ | 90°6′57" | 1218.5 | H11 | 1.92 | 60 mL |
| FCM-12 | Qitai County, Changji | Han Gou Group 2 | 44°10'19" | 89°23′51" | 664.9 | H12 | 2.48 | 60 mL |
| FCM-13 | Saiyibak District, Urumqi | Quanyin Qishan Camping Reso | 43°41′36.9″ | 87°23′59.4″ | 1030 | H13 | 3.16 | 60 mL |
| FCM-14 | Dabancheng District, Urumqi | Three Mountains | 43°30'5" | 88°53'6" | 1178 | H14 | 2.16 | 60 mL |
| FCM-15 | Midong District, Urumqi | Xishan | 43°31′30″ | 87°20′37″ | 1150 | H15 | 2.13 | 60 mL |
| CM | Shuimogou District, Urumqi | Gejiagou, Shiren Gou Subdistrict | 43°48'48" | 87°44'2" | 865 | H16 | 6.89 | 20 Kg |

**Table S2. Molecular identification and sequence characteristics of representative LAB and yeast isolates.**

| Isolate | Group | Final identification | Marker | Isolation source | Sequence length (bp) | Closest BLAST match | Identity (%) | Query coverage (%) | Reference accession | GenBank accession |
|---|---|---|---|---|---|---|---|---|---|---|
| DL1 | LAB | Lactococcus lactis | 16S rRNA | FCM-14 (Urumqi) | 1419 | Lactococcus lactis strain T32-1 | 99.366 | 100.00 | PQ835559.1 | QB030565 |
| DL2 | LAB | Lactococcus lactis | 16S rRNA | FCM-2 (Altay) | 1417 | Lactococcus lactis strain Sourdough_H17 | 100.000 | 100.00 | OL823008.1 | QB030579 |
| DL3 | LAB | Lactobacillus helveticus | 16S rRNA | FCM-7 (Aksu) | 1439 | Lactobacillus helveticus strain NWAFU1518 | 99.931 | 100.00 | MG551186.1 | QB030569 |
| DL4 | LAB | Streptococcus thermophilus | 16S rRNA | FCM-1 (Altay) | 1428 | Streptococcus thermophilus strain MLHL-7 | 100.000 | 100.00 | EU419603.1 | QB030593 |
| DL5 | LAB | Lactococcus lactis | 16S rRNA | FCM-3 (Altay) | 1414 | Lactococcus lactis strain 4477 | 99.646 | 100.00 | OL823008.1 | QB030564 |
| DL6 | LAB | Lacticaseibacillus paracasei | 16S rRNA | FCM-13 (Urumqi) | 1488 | Lacticaseibacillus paracasei strain 10266 | 100.000 | 99.73 | HE983621.1 | QB030595 |
| DL7 | LAB | Lactiplantibacillus plantarum | 16S rRNA | FCM-1 (Altay) | 1433 | Lactiplantibacillus plantarum strain P6 | 99.721 | 100.00 | OM301983.1 | QB030583 |

| | | | | | | | | | | |
|---|---|---|---|---|---|---|---|---|---|---|
| DL8 | LAB | Lactococcus lactis | 16S rRNA | FCM-12 (Changji) | 1417 | Lactococcus lactis strain MG5129 | 99.506 | 100.00 | OL823008.1 | QB030560 |
| DL9 | LAB | Lactococcus lactis subsp. lactis | 16S rRNA | FCM-2 (Altay) | 1334 | Lactococcus lactis subsp. lactis LM 0404 | 99.551 | 100.00 | OQ406106.1 | QB030584 |
| DL10 | LAB | Lactiplantibacillus pentosus | 16S rRNA | FCM-8 (Aksu) | 1438 | Lactiplantibacillus pentosus strain S6 | 99.931 | 100.00 | MW618651.1 | QB030575 |
| DL11 | LAB | Streptococcus thermophilus | 16S rRNA | FCM-9 (Aksu) | 1422 | Streptococcus thermophilus strain TW10-3 | 100.000 | 99.79 | MT538720.1 | QB030594 |
| DL12 | LAB | Lactiplantibacillus plantarum | 16S rRNA | FCM-15 (Urumqi) | 1482 | Lactiplantibacillus plantarum strain SRCM102022 | 99.933 | 100.00 | CP123053.1 | QB030576 |
| DL13 | LAB | Lactococcus lactis | 16S rRNA | FCM-11 (Changji) | 1413 | Lactococcus lactis strain HNL12 | 99.929 | 100.00 | OL823008.1 | QB030559 |
| DL14 | LAB | Lacticaseibacillus paracasei | 16S rRNA | FCM-10 (Changji) | 1478 | Lacticaseibacillus paracasei strain VHProbi F22 | 99.932 | 100.00 | CP195898.1 | QB030572 |
| DL15 | LAB | Streptococcus thermophilus | 16S rRNA | FCM-2 (Altay) | 1426 | Streptococcus thermophilus strain YNDY-3 | 100.000 | 100.00 | MT544658.1 | QB030589 |
| DL16 | LAB | Lactiplantibacillus pentosus | 16S rRNA | FCM-4 (Ili) | 1446 | Lactiplantibacillus pentosus strain HBUAS51907 | 100.000 | 100.00 | ON025645.1 | QB030561 |
| DL17 | LAB | Lactococcus lactis | 16S rRNA | FCM-6 (Ili) | 1417 | Lactococcus lactis strain DHC-8 | 99.788 | 100.00 | OL823008.1 | QB030574 |

| | | | | | | | | | | |
|---|---|---|---|---|---|---|---|---|---|---|
| DL18 | LAB | Streptococcus thermophilus | 16S rRNA | FCM-7 (Aksu) | 1424 | Streptococcus thermophilus strain FMA808 | 100.000 | 100.00 | OP714512.1 | QB030581 |
| DL19 | LAB | Lacticaseibacillus paracasei | 16S rRNA | FCM-12 (Changji) | 1492 | Lacticaseibacillus paracasei strain XM-38 | 99.933 | 100.00 | CP195898.1 | QB030597 |
| DL20 | LAB | Lactococcus lactis subsp. lactis | 16S rRNA | FCM-13 (Urumqi) | 1417 | Lactococcus lactis subsp. lactis strain UC06 | 100.000 | 100.00 | MT597537.1 | QB030587 |
| DL21 | LAB | Lacticaseibacillus paracasei | 16S rRNA | FCM-3 (Altay) | 1486 | Lacticaseibacillus paracasei strain SDCM 1102 | 100.000 | 99.60 | MW205775.1 | QB030588 |
| DL22 | LAB | Lacticaseibacillus paracasei | 16S rRNA | FCM-7 (Aksu) | 1486 | Lacticaseibacillus paracasei strain IMUST00467 | 99.933 | 100.00 | PV382627.1 | QB030592 |
| DL23 | LAB | Streptococcus thermophilus | 16S rRNA | FCM-1 (Altay) | 1431 | Streptococcus thermophilus strain FMA408 | 100.000 | 100.00 | MF893892.1 | QB030582 |
| DL24 | LAB | Lactiplantibacillus argentoratensis | 16S rRNA | FCM-6 (Ili) | 1482 | Lactiplantibacillus argentoratensis strain 12-27B | 99.865 | 100.00 | CP193775.1 | QB030571 |
| DL25 | LAB | Lactococcus lactis | 16S rRNA | FCM-5 (Ili) | 1416 | Lactococcus lactis strain 4596 | 100.000 | 100.00 | MT544908.1 | QB030566 |
| DL26 | LAB | Lactiplantibacillus plantarum | 16S rRNA | FCM-13 (Changji) | 1008 | Lactobacillus plantarum strain LM3-8 | 98.902 | 99.31 | PX904960.1 | QB030570 |

| | | | | | | | | | | |
|---|---|---|---|---|---|---|---|---|---|---|
| DL27 | LAB | Lactobacillus helveticus | 16S rRNA | FCM-10 (Changji) | 1438 | Lactobacillus helveticus strain NWAFU1516 | 99.861 | 100.00 | MG551184.1 | QB030567 |
| DL28 | LAB | Lacticaseibacillus paracasei subsp. tolerans | 16S rRNA | FCM-3 (Altay) | 1488 | Lacticaseibacillus paracasei subsp. tolerans strain MGB0734 | 100.000 | 100.00 | CP195898.1 | QB030585 |
| DL29 | LAB | Lactiplantibacillus plantarum | 16S rRNA | FCM-5 (Ili) | 1468 | Lactiplantibacillus plantarum strain SRCM210576 | 100.000 | 100.00 | CP123053.1 | QB030586 |
| DL30 | LAB | Streptococcus thermophilus | 16S rRNA | FCM-9 (Aksu) | 1426 | Streptococcus thermophilus strain BURD PB4 | 100.000 | 100.00 | MT544730.1 | QB030596 |
| DL31 | LAB | Leuconostoc garlicum | 16S rRNA | FCM-13 (Urumqi) | 1467 | Leuconostoc garlicum strain L1 | 100.000 | 99.45 | DQ239691.1 | QB030580 |
| DL32 | LAB | Lactiplantibacillus plantarum | 16S rRNA | FCM-15 (Urumqi) | 1006 | Lactiplantibacillus plantarum strain S1 | 99.391 | 98.01 | OL719063.1 | QB030573 |
| DL33 | LAB | Lacticaseibacillus rhamnosus | 16S rRNA | FCM-9 (Aksu) | 1444 | Lacticaseibacillus rhamnosus strain Y2-K-1 | 100.000 | 100.00 | CP195898.1 | QB030577 |
| DL34 | LAB | Lactobacillus kefiri | 16S rRNA | FCM-11 (Changji) | 1428 | Lactobacillus kefiri strain 4618 | 99.930 | 99.86 | MT459360.1 | QB030568 |
| DL35 | LAB | Enterococcus italicus | 16S rRNA | FCM-8 (Aksu) | 1439 | Enterococcus italicus strain FMA295 | 99.931 | 100.00 | HQ721276.1 | QB030591 |

| | | | | | | | | | | |
|---|---|---|---|---|---|---|---|---|---|---|
| DL36 | LAB | Lactococcus lactis | 16S rRNA | FCM-14 (Urumqi) | 1423 | Lactococcus lactis strain A5686 | 100.000 | 100.00 | OQ775345.1 | QB030590 |
| DL37 | LAB | Enterococcus pseudoavium | 16S rRNA | FCM-5 (Ili) | 1424 | Enterococcus pseudoavium strain EP | 99.159 | 100.00 | NR_028705.1 | QB030562 |
| DL38 | LAB | Lactococcus lactis | 16S rRNA | FCM-5 (Ili) | 1417 | Lactococcus lactis strain 4469 | 99.859 | 100.00 | OL823008.1 | QB030563 |
| DL39 | LAB | Lacticaseibacillus rhamnosus | 16S rRNA | FCM-12 (Changji) | 1443 | Lacticaseibacillus rhamnosus strain 16469 | 100.000 | 100.00 | MW931816.1 | QB030578 |
| DY1 | Yeast | Pichia kudriavzevii | 26S rDNA D1/D2 | FCM-7 (Aksu) | 610 | Pichia kudriavzevii isolate Sample33_2-144 | 99.834 | 98.52 | OR250116.1 | QB030467 |
| DY2 | Yeast | Kluyveromyces marxianus | 26S rDNA D1/D2 | FCM-6 (Ili) | 594 | Kluyveromyces marxianus KDLYH2-1 | 100.000 | 100.00 | KF633180.1 | QB030477 |
| DY3 | Yeast | Kluyveromyces marxianus | 26S rDNA D1/D2 | FCM-4 (Ili) | 576 | Kluyveromyces marxianus isolate U-MF10 | 100.000 | 99.48 | KM279378.1 | QB030473 |
| DY4 | Yeast | Saccharomyces cerevisiae | 26S rDNA D1/D2 | FCM-11 (Changji) | 590 | Saccharomyces cerevisiae strain SYM23-2 26S | 99.831 | 100.00 | MN462947.1 | QB030460 |
| DY5 | Yeast | Saccharomyces cerevisiae | 26S rDNA D1/D2 | FCM-1 (Altay) | 584 | Saccharomyces cerevisiae isolate S1 | 99.829 | 100.00 | AP045000.1 | QB030459 |

| | | | | | | | | | | |
|---|---|---|---|---|---|---|---|---|---|---|
| DY6 | Yeast | Saccharomyces cerevisiae | 26S rDNA D1/D2 | FCM-3 (Altay) | 592 | Saccharomyces cerevisiae strain TY12 | 100.000 | 99.66 | MW969694.1 | QB030453 |
| DY7 | Yeast | Kluyveromyces marxianus | 26S rDNA D1/D2 | FCM-9 (Aksu) | 576 | Kluyveromyces marxianus strain EX24C4 | 99.825 | 99.48 | AY894820.1 | QB030469 |
| DY8 | Yeast | Kluyveromyces marxianus | 26S rDNA D1/D2 | FCM-14 (Urumqi) | 571 | Kluyveromyces marxianus isolate 2-1-4 | 100.000 | 100.00 | PV491872.1 | QB030471 |
| DY9 | Yeast | Saccharomyces cerevisiae | 26S rDNA D1/D2 | FCM-14 (Urumqi) | 582 | Saccharomyces cerevisiae isolate TTH6 | 100.000 | 100.00 | ON775518.1 | QB030461 |
| DY10 | Yeast | Nakaseomyces glabratus | 26S rDNA D1/D2 | FCM-12 (Changji) | 614 | Nakaseomyces glabratus isolate SY1-1 | 100.000 | 99.19 | MF148859.1 | QB030468 |
| DY11 | Yeast | Kluyveromyces marxianus | 26S rDNA D1/D2 | FCM-2 (Altay) | 579 | Kluyveromyces marxianus isolate KDLYH2-1 | 100.000 | 98.96 | MW969707.1 | QB030463 |
| DY12 | Yeast | Saccharomyces cerevisiae | 26S rDNA D1/D2 | FCM-10 (Changji) | 587 | Saccharomyces cerevisiae isolate S1 | 99.830 | 100.00 | MN462938.1 | QB030454 |
| DY13 | Yeast | Kazachstania unispora | 26S rDNA D1/D2 | FCM-8 (Aksu) | 730 | Kazachstania unispora strain DA-57 | 99.587 | 99.45 | KX376257.1 | QB030476 |

| | | | | | | | | | | |
|---|---|---|---|---|---|---|---|---|---|---|
| DY14 | Yeast | Kluyveromyces marxianus | 26S rDNA D1/D2 | FCM-6 (Ili) | 583 | Kluyveromyces marxianus isolate KDLYH4-1 | 99.314 | 100.00 | KF633182.1 | QB030466 |
| DY15 | Yeast | Pichia kudriavzevii | 26S rDNA D1/D2 | FCM-9 (Aksu) | 606 | Pichia kudriavzevii strain ZJ-11 | 99.504 | 99.83 | PZ459058.1 | QB030465 |
| DY16 | Yeast | Kluyveromyces marxianus | 26S rDNA D1/D2 | FCM-8 (Aksu) | 572 | Kluyveromyces marxianus isolate 2-8-3 | 99.825 | 100.00 | MW969707.1 | QB030474 |
| DY17 | Yeast | Kazachstania unispora | 26S rDNA D1/D2 | FCM-11 (Changji) | 586 | Kazachstania unispora culture CBS:2422 | 99.829 | 100.00 | OZ188223.1 | QB030462 |
| DY18 | Yeast | Saccharomyces cerevisiae | 26S rDNA D1/D2 | FCM-13 (Urumqi) | 596 | Saccharomyces cerevisiae strain JBY-11 | 99.664 | 100.00 | AP045000.1 | QB030456 |
| DY19 | Yeast | Torulaspora delbrueckii | 26S rDNA D1/D2 | FCM-2 (Altay) | 581 | Torulaspora delbrueckii culture CBS:813 | 100.000 | 99.83 | MT449110.1 | QB030458 |
| DY20 | Yeast | Kazachstania unispora | 26S rDNA D1/D2 | FCM-1 (Altay) | 568 | Kazachstania unispora isolate PYCC 2500 | 100.000 | 99.65 | MW316683.1 | QB030475 |
| DY21 | Yeast | Torulaspora delbrueckii | 26S rDNA D1/D2 | FCM-6 (Ili) | 586 | Torulaspora delbrueckii culture CBS:813 | 99.829 | 99.83 | PX453190.1 | QB030457 |

| | | | | | | | | | | |
|---|---|---|---|---|---|---|---|---|---|---|
| DY22 | Yeast | Pichia kudriavzevii | 26S rDNA D1/D2 | FCM-11 (Changji) | 594 | Pichia kudriavzevii isolate YT4 | 99.831 | 99.66 | PP916644.1 | QB030464 |
| DY23 | Yeast | Kazachstania unispora | 26S rDNA D1/D2 | FCM-15 (Urumqi) | 607 | Kazachstania unispora strain CZNJM-3 | 99.506 | 100.00 | OZ188223.1 | QB030472 |
| DY24 | Yeast | Kluyveromyces marxianus | 26S rDNA D1/D2 | FCM-9 (Aksu) | 571 | Kluyveromyces marxianus isolate 2-1-4 | 99.825 | 100.00 | PV491872.1 | QB030470 |
| DY25 | Yeast | Saccharomyces cerevisiae | 26S rDNA D1/D2 | FCM-5 (Ili) | 583 | Saccharomyces cerevisiae isolate S1 | 99.828 | 100.00 | AP045000.1 | QB030455 |

**Table S3. The polydispersity index (PDI) of LAB and yeast-fermented milk.**

| Strain/Sample | Group | PDI (mean ± SD) |
|---|---|---|
| MC | LAB | 0.241 ± 0.025 |
| DL37 | LAB | 0.492 ± 0.009 |
| DL29 | LAB | 0.468 ± 0.015 |
| DL21 | LAB | 0.322 ± 0.008 |
| DL18 | LAB | 0.599 ± 0.035 |
| DL6 | LAB | 0.242 ± 0.023 |
| DL19 | LAB | 0.260 ± 0.014 |
| DL23 | LAB | 0.618 ± 0.042 |
| DY5 | Yeast | 0.399 ± 0.047 |
| DY23 | Yeast | 0.326 ± 0.014 |
| DY3 | Yeast | 0.171 ± 0.016 |
| DY9 | Yeast | 0.165 ± 0.044 |
| DY2 | Yeast | 0.221 ± 0.027 |

**Table S4. Genomic characteristics and safety-related features of the selected strains DL19, DL29, and DY3.**

| Strain | Genome size | GC | Scaffolds | N50 | Proteins | CARD raw hits | Retained acquired AMR determinants | VFDB/DFVF/PHI interpretation |
|---|---|---|---|---|---|---|---|---|
| DL19 | 3.00 Mb | 46.19% | 220 | 58.7 kb | 3,198 | 70 | Not identified | No major VFDB determinant retained |
| DL29 | 3.19 Mb | 44.36% | 140 | 51.6 kb | 3,241 | 79 | Not identified | No major VFDB determinant retained |
| DY3 | 11.70 Mb | 39.62% | 3,468 | 33.9 kb | 6,284 | — | — | DFVF/PHI hits interpreted as contextual homologs |
